# Microbiome-derived metabolites shape CD4^+^ T cell differentiation and immune aging in HIV-1 infection

**DOI:** 10.1172/jci.insight.204383

**Published:** 2026-07-21

**Authors:** Amanda Cabral Da Silva, Luke Flantzer, Jaclyn Weinberg, Shuya Kyu, Lisa P. Daley-Bauer, Anyce Godoy, Ana Carolina Santana, Aarthi Talla, Amber Lynn Rittgers, Sarah Welbourn, David Ezra Gordon, Jeffery Alan Tomalka, Vincent C. Marconi, Dean P. Jones, Souheil-Antoine Younes

**Affiliations:** 1 Pathology Advanced Translational Research Unit (PATRU), Department of Pathology and Laboratory Medicine, and; 2 Division of Pulmonary, Allergy, Critical Care and Sleep Medicine, Department of Medicine, Emory University School of Medicine, Atlanta, Georgia, USA.; 3 IMMUNASYST, Scottsdale, Arizona, USA.; 4 Division of Infectious Diseases Emory University School of Medicine and Department of Global Health, Emory University Rollins School of Public Health, Atlanta, Georgia, USA.

**Keywords:** AIDS/HIV, Aging, Immunology, Adaptive immunity, Cellular senescence, Metabolomics

## Abstract

The role of aromatic gut-derived bacterial metabolites (GDBMs) in shaping immune cell metabolism and function remains poorly explored. Using ex vivo metabolomic profiling of paired plasma and CD4^+^ T cells from people living with HIV-1 (PLWH), we identified a network of aromatic GDBMs whose cell-associated abundance, rather than systemic levels, was linked to broad alterations in CD4^+^ T cell metabolic and functional states. Among these, p-cresol sulfate (PCS) emerged as a mechanistic prototype. Ex vivo flow cytometry and scRNA-seq of CD4^+^ T cells stratified by cell-associated PCS levels revealed dose-dependent enrichment of transcriptional programs associated with impaired differentiation, regulatory-like identity, and cellular senescence. In vitro transcriptomic and proteomic analyses of PCS-exposed CD4^+^ T cells demonstrated induction of cell-cycle arrest, mitochondrial dysfunction, and senescence-associated programs, including upregulation of p16 and p21. Integration of these immunometabolic findings with HIV-1 reservoir measurements revealed that CD4^+^ T cell states defined by cell-associated GDBMs track with intact proviral DNA levels in vivo. These findings define a microbiome-derived axis that reshapes CD4^+^ T cell metabolism and fate, promotes immune aging in PLWH, and may foster immunometabolic states linked to long-term HIV-1 reservoir persistence.

## Abstract 2

Gut bacteria produce toxins that accumulate inside immune cells and accelerate aging revealing a new mechanism of immune dysfunction in HIV infection.

## Graphical abstract

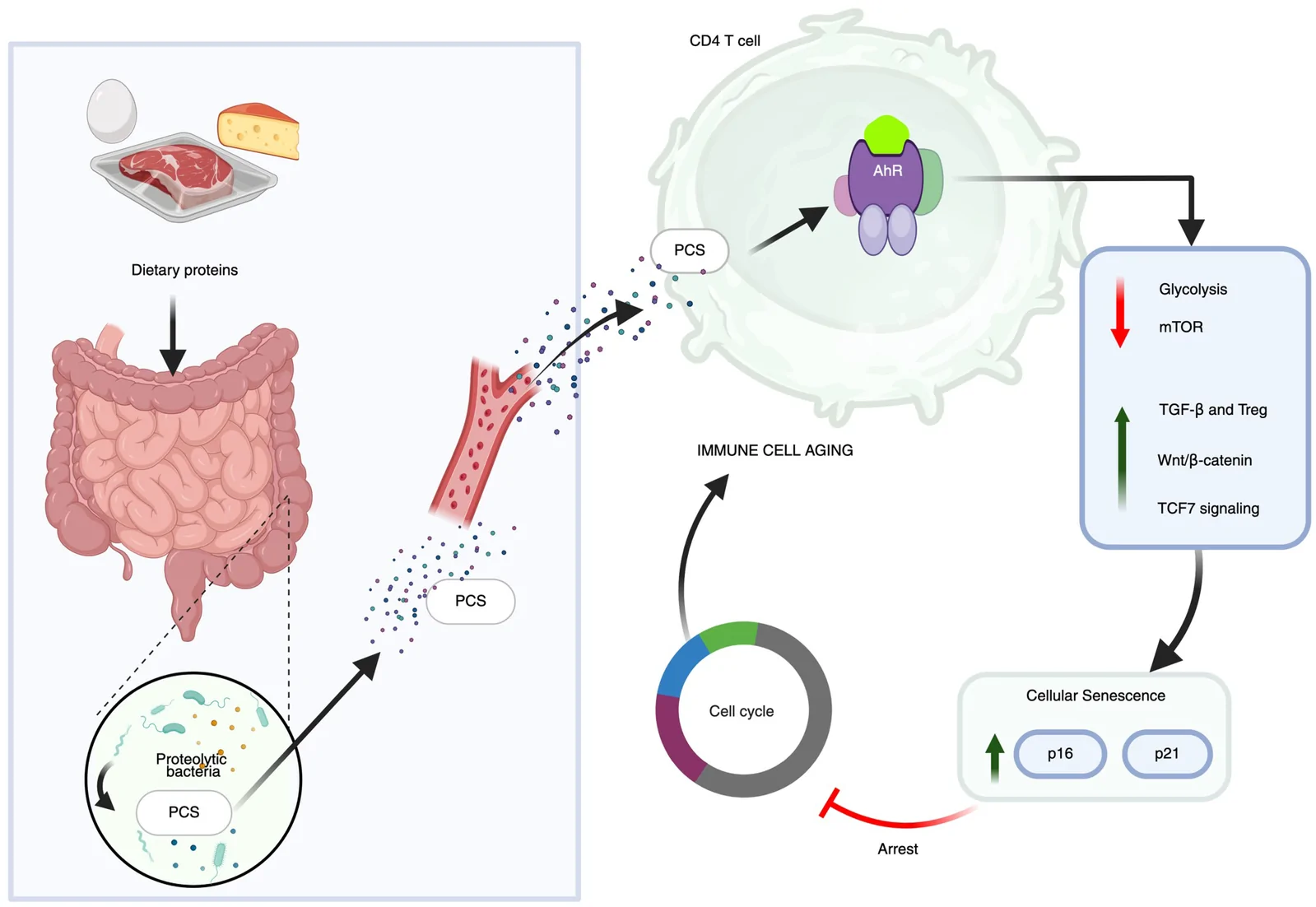

## Introduction

Gut-derived bacterial metabolites (GDBMs) are increasingly recognized as key modulators of host immunity and metabolic function. While short-chain fatty acids (SCFAs) (1–3) and microbiota-modified secondary bile acids (4, 5) are well characterized for their roles in immunity, less is known about the effects of other GDBMs, particularly aromatic metabolites such as p-cresol sulfate (PCS), p-cresol glucuronide (PCG), phenylacetylglycine (PAG), and indole-3-acetic acid (IAA), which are generated through bacterial degradation of amino acids by the gut bacterial flora (6). These metabolites can reach peripheral tissues to exert immunomodulatory effects, but their functional impact on human T cells remains largely unexplored. Among these, PCS, a prototypical GDBM, accumulates in circulation during conditions of dysbiosis, chronic inflammation, and renal dysfunction (6–9). PCS originates from bacterial degradation of tyrosine and phenylalanine in the gut, is sulfated in the liver, and is normally cleared by the kidney (7). Elevated PCS levels are associated with oxidative stress (10), endothelial dysfunction (11), and impaired immune responses in chronic disease states (12, 13). However, the cell-intrinsic effects of PCS on adaptive immunity, particularly CD4^+^ T cell function and fate, remain poorly defined. We previously investigated the role of PCS in people living with HIV-1 (PLWH), focusing on individuals who fail to restore CD4^+^ T cell counts despite effective antiretroviral therapy (ART), Immune nonresponders (INR). In that study, PCS levels were elevated in both plasma and CD4^+^ T cells and negatively correlated with CD4^+^ T cell counts. In vitro exposure of healthy CD4^+^ T cells to PCS impaired proliferation and altered mitochondrial function, suggesting that this metabolite may contribute to immune dysfunction in PLWH. In addition, microbiome analyses revealed enrichment of bacterial taxa capable of producing PCS in stool samples of INR (8).

CD4^+^ T cells are central orchestrators of adaptive immunity, integrating environmental cues to coordinate effector, memory, and regulatory responses. Their activation and differentiation are tightly coupled to metabolic reprogramming, with key pathways such as mTOR, glycolysis, and oxidative phosphorylation supporting lineage commitment and effector function (14–16). Disruption of these metabolic circuits can compromise T cell fate decisions, promote dysfunction, or lead to the emergence of stem-like or exhausted states, particularly under conditions of chronic antigen exposure or metabolic stress (17). Senescence in CD4^+^ T cells, characterized by cell cycle arrest, loss of proliferative capacity, and altered metabolic programs, is increasingly recognized as a feature of both immunological aging and chronic infections (18–20). This phenotype is associated with mitochondrial dysfunction, altered redox balance, and the expression of canonical regulators such as p16 and p21 proteins (18, 21).

HIV-1 infection accelerates immune cell aging through multiple mechanisms, many of which mirror natural immunosenescence observed in elderly individuals (22, 23). Chronic immune activation is a hallmark of HIV infection, driving repeated cycles of immune cell proliferation, activation, and turnover that ultimately exhaust regenerative capacity (24, 25). CD4^+^ T cells undergo replicative senescence, reduced proliferative potential, increased expression of senescence markers such as p16 (26). Together, these processes compromise immune homeostasis and diminish the capacity to respond to new antigens, contributing to the premature immune aging observed in PLWH (27–30).

Several studies have shown that metabolic features of CD4^+^ T cells influence HIV-1 reservoir seeding and persistence, with glycolytic and oxidative phosphorylation activity, mTOR signaling, and lipid metabolism all implicated as determinants of reservoir size and CD4^+^ T cell susceptibility to infection (31–40). Despite these advances, the upstream metabolic triggers that shape reservoir-permissive CD4^+^ T cell states remain largely undefined.

The effect of GDBMs on the metabolic programming and fate decisions of CD4^+^ T cells in HIV-1 infection settings, including immune aging, differentiation, senescence, and functional capacity, remains largely unexplored. Here, we combine multi-omics profiling, including transcriptomics, proteomics, cytokine analysis, and metabolomics, with functional in vitro assays and ex vivo validation in PLWH to dissect the effects of PCS on CD4^+^ T cell biology. We demonstrated that PCS exposure drives CD4^+^ T cell senescence and promotes a regulatory-like transcriptional program, while concurrently restraining effector differentiation. PCS represses mTOR and effector cytokine programs while activating TGF-β, the aryl hydrocarbon receptor (AhR), and Wnt signaling, and reshapes the T cell proteome toward mitochondrial stress and metabolic adaptation. PCS exemplifies a microbial metabolite that reshapes CD4^+^ T cell fate through metabolic and transcriptional reprogramming and may contribute to an immune environment conducive to HIV-1 reservoir persistence.

## Results

### Activation-dependent cell association of PCS in CD4^+^ T cells is independent of plasma levels.

To determine whether PCS levels in plasma reflect cell association in CD4^+^ T cells, we quantified PCS the concentrations in matched plasma and sorted CD4^+^ T cells from 50 PLWH. Mass spectrometry analysis revealed that cell-associated PCS levels were significantly lower than those in plasma, with mean concentrations of 0.025 μM in CD4^+^ T cells versus 11.8 μM in plasma (*P* < 0.0001) (Supplemental Figure 1A; supplemental material available online with this article; https://doi.org/10.1172/jci.insight.204383DS1). Despite this large disparity, no significant correlation was observed between plasma and cell-associated PCS levels (Spearman r = –0.1861, *P* = 0.1957) (Supplemental Figure 1B), suggesting that circulating PCS levels do not reliably predict CD4^+^ T cell association. To further investigate the kinetics and determinants of PCS cell association, CD4^+^ T cells from healthy donors were incubated with 50 or 100 μM PCS for 1, 3, or 6 days. PCS uptake was detectable at all time points and was dose dependent, with cell-association concentrations ranging from 4 to 8 nM at 50 μM PCS and ~8–16 nM at 100 μM PCS (Supplemental Figure 1C). Notably, T cell receptor (TCR) stimulation with anti-CD3/CD28 during PCS exposure on day 6 enhanced PCS cell association. Together, these data demonstrate that PCS associates with CD4^+^ T cells in a dose- and activation-dependent manner, independent of circulating protein-bound PCS concentrations, which fail to predict CD4^+^ T cell–associated PCS levels. Importantly, exposure to 100 μM PCS results in quantifiable nanogram-scale levels of cell-associated PCS in CD4^+^ T cells.

### Cell-associated GDBMs in CD4^+^ T cells from PLWH associate with TCA cycle disruption and global metabolic dysregulation.

**Figure 1:**
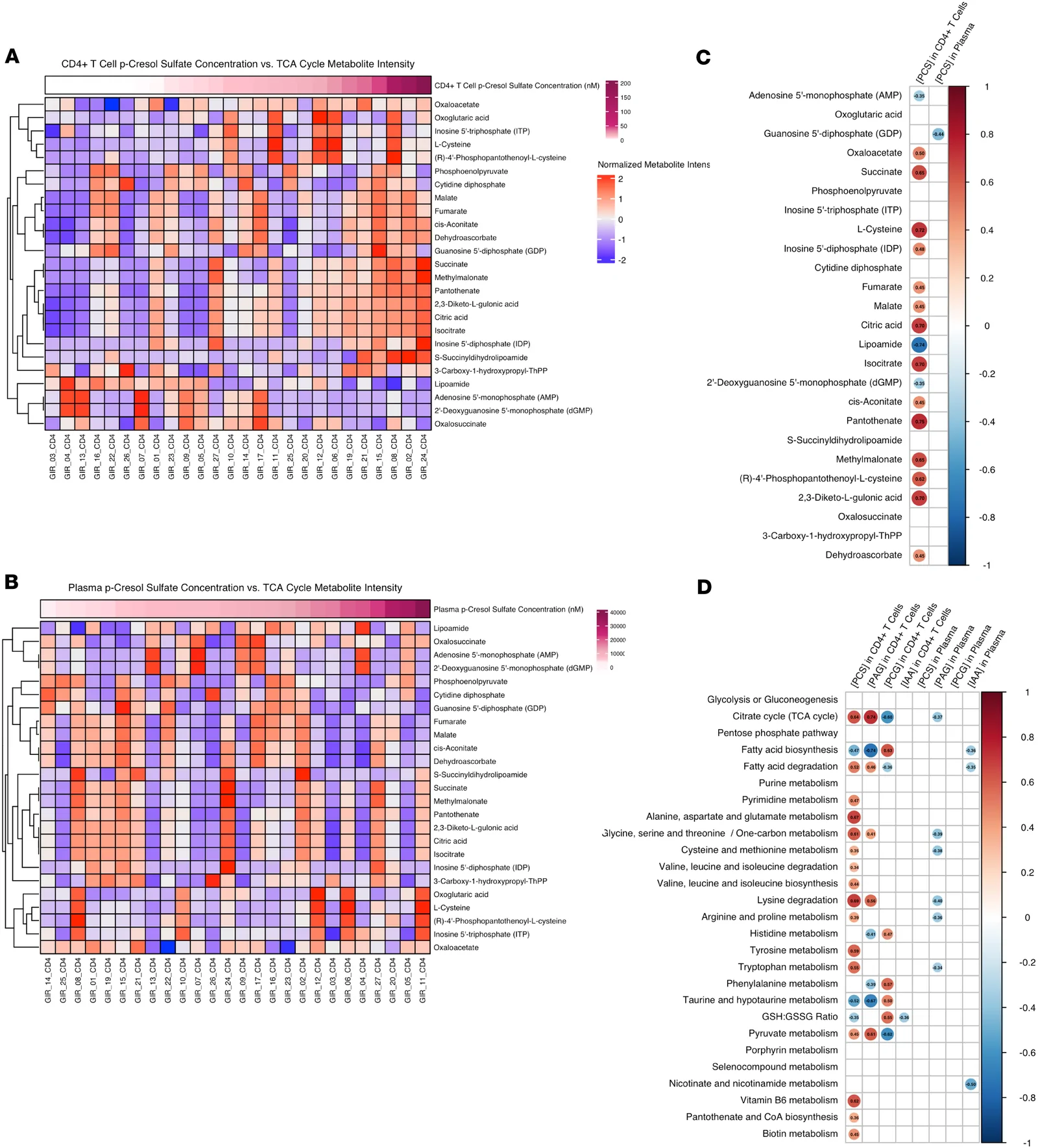
Metabolite Profiling and PCS Quantification in CD4^+^ T cells from PLWH. CD4^+^ T cells were sorted from PBMCs of 26 immune responders PLWH. Cell-associated and plasma concentrations PCS were quantified by targeted mass spectrometry. Untargeted metabolomics analysis was performed on sorted CD4^+^ T cells using high-resolution LC-MS. (**A** and **B**) Normalized intensities of tricarboxylic acid (TCA) cycle metabolites stratified by cell-associated PCS (**A**) or plasma PCS (**B**) concentrations. (**C**) Spearman correlation coefficients were calculated between cell-associated PCS levels and individual TCA metabolites. (**D**) Spearman correlation heatmap showing associations between the concentrations of PCS, PAG, PCG, and IAA measured in CD4^+^ T cells or plasma, and the enrichment of metabolic pathways identified by untargeted metabolomics. Rows represent metabolic pathways, and columns represent cell–associated or plasma metabolite levels. Circle size and color reflect the magnitude and direction of the correlation (red, positive; blue, negative). Metabolite comparisons were analyzed using FDR correction for multiple testing, with FDR-adjusted *P* < 0.05 considered statistically significant.

We then performed untargeted metabolomics along with cell-associated PCS, PAG, PCG, and IAA quantification by targeted metabolic analysis. Metabolic profiling was performed on lysate of CD4^+^ T cells from 26 immune-responders PLWH (listed below Figure 1, A and B). As shown in Figure 1A, we observed a gradient of cell-associated PCS concentrations across individuals that significantly stratified the abundance of multiple tricarboxylic acid (TCA) cycle intermediates. Higher cell-associated PCS levels were associated with elevated levels of key metabolites such as citrate, α-ketoglutarate, succinate, malate, and fumarate, suggesting accumulation of incompletely oxidized TCA intermediates consistent with mitochondrial dysfunction. In contrast, plasma PCS concentrations did not show the same degree of association with TCA metabolites (Figure 1B), indicating that cell-associated PCS levels, not circulating levels, are more reflective of T cell metabolic perturbation. Then, Spearman correlation analysis (Figure 1C) confirmed that PCS levels within CD4^+^ T cells were positively correlated with several central TCA metabolites, including AMP, succinate, and fumarate, further supporting PCS-associated bioenergetic stress and mitochondrial inefficiency. To extend beyond TCA cycle alterations, we used untargeted metabolomics to assess how PCS cell-level and related GDBMs (PAG, PCG, and IAA) modulate broader metabolic pathway activity in CD4^+^ T cells. In Figure 1D, cell-level PCS showed the broadest and most coherent associations with CD4^+^ T cell metabolism. Higher CD4^+^ T cell–associated PCS concentrations positively correlated with multiple core pathways — most prominently central carbon/energy and biosynthetic programs, including the TCA cycle, glycolysis/gluconeogenesis, purine and pyrimidine metabolism, and several amino-acid catabolic routes. PCS also correlated with pathways related to redox balance and cofactor metabolism, consistent with broad metabolic remodeling linked to PCS accumulation. Importantly, these pathway-level correlations were detected for cell-associated PCS, reinforcing that cellular retention, not plasma abundance, aligns with metabolic remodeling. Expanding beyond PCS, PAG displayed a correlation pattern that largely mirrored PCS. Cell-associated PAG showed a similar metabolic footprint to PCS, positively correlating with pathways related to energy production, nucleotide biosynthesis, and amino acid processing. This similarity suggests that PCS and PAG participate in a shared aromatic GDBM-associated metabolic network within CD4^+^ T cells, with both metabolites tracking along the same direction of pathway perturbation. By contrast, PCG showed an opposing relationship to this PCS/PAG axis. Cell-associated PCG concentrations correlate negatively with several of the same pathways that rose with PCS and PAG, including energy-linked and biosynthetic programs. These inverse associations position PCG within the same aromatic GDBM family yet suggest a metabolically divergent role in CD4^+^ T cell regulation, implying heterogeneity in how structurally related GDBMs map onto CD4^+^ T cell metabolic states. Finally, IAA did not display significant associations with CD4^+^ T cell metabolic pathways in this dataset.

Together, these findings demonstrate that cell-associated accumulation of specific aromatic GDBMs, particularly PCS and PAG, is tightly linked to global metabolic reprogramming in CD4^+^ T cells from PLWH. PCS exhibited the strongest and most consistent associations with disrupted energy metabolism and biosynthetic pathways, reinforcing its role as a key immunometabolic effector. Of the 27 metabolic pathways assessed, cell-associated PCS concentrations correlated with 19 pathways, reflecting the broadest metabolic effect among the GDBMs analyzed. PAG correlated with 9 pathways, displaying partial overlap with PCS, while PCG was associated with 8 pathways, often in an opposing direction to PCS and PAG. Importantly, these metabolic alterations were specific to cell-associated GDBM abundance and were not reflected by plasma concentrations, underscoring the importance of considering cellular association when assessing the immunometabolic effect of GDBMs.

The kynurenine/tryptophan (K/T) ratio reflects the activity of indoleamine 2,3-dioxygenase 1 (IDO1), the rate-limiting enzyme that catabolizes tryptophan into kynurenine along the kynurenine pathway (41, 42). IDO1 activity is induced by chronic immune activation and has been shown to be elevated in PLWH, where a high K/T ratio is associated with T cell dysfunction, disease progression, and poor immune restoration despite ART (43–45). To investigate whether intracellular PCS accumulation in CD4^+^ T cells is linked to tryptophan catabolism, we examined the correlation between PCS levels and the K/T ratio in our cohort. Plasma PCS showed no significant correlation with the K/T ratio (*r* = 0.025, *P* = 0.904, *n* = 26; Supplemental Figure 2A). In contrast, intracellular PCS concentrations in CD4^+^ T cells were significantly and inversely correlated with the K/T ratio (*r* = –0.530, *P* = 0.005, *n* = 26; Supplemental Figure 2B), suggesting that intracellular PCS and the K/T ratio are mutually exclusive in CD4^+^ T cells.

### Cell-associated GDBMs correlate with metabolic pathways linked to the intact HIV-1 reservoir.

**Figure 2:**
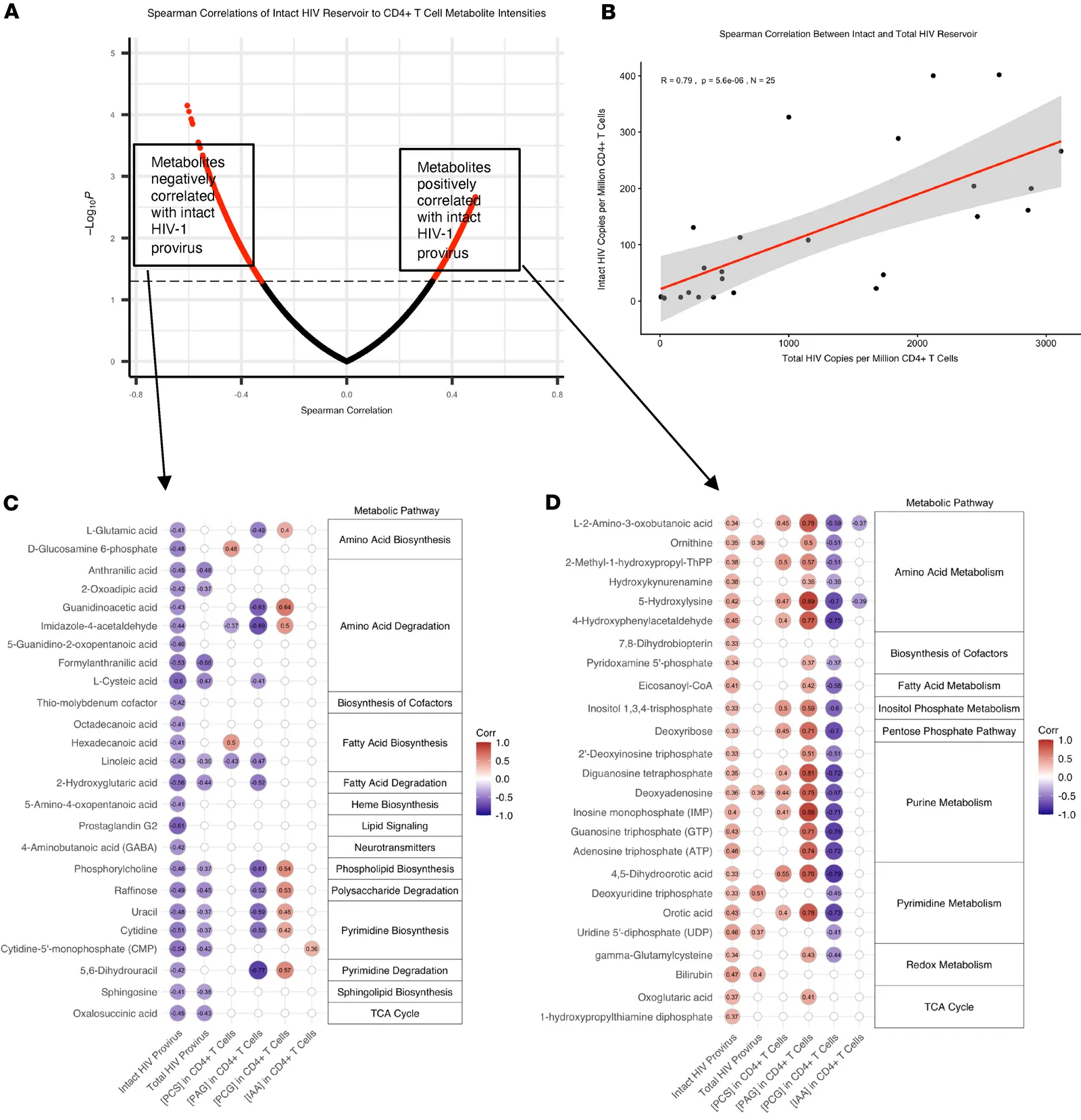
Metabolic pathway correlations with intact and total HIV-1 reservoir levels in CD4^+^ T cells and relationship to the GDBMs. (**A**) Volcano plot showing Spearman correlations between CD4^+^ T cell metabolite intensities and intact HIV-1 reservoir size. Red points indicate statistically significant correlations (*P* < 0.05), and boxed regions highlight metabolites with strong negative (left) and positive (right) associations. (**B**) Linear regression of intact versus total HIV-1 copies per million CD4^+^ T cells, demonstrating a strong positive correlation (R = 0.79, *P* = 5.6 × 10 ^–6^, *n* = 25). (**C**) Heatmap of negatively correlated metabolites (highlighted in **A**, left box), categorized by metabolic pathways. (**D**) Heatmap of positively correlated metabolites (highlighted in **A**, right box), with pathway annotations. Circle size and color scale represent strength and direction of Spearman correlation coefficients between each metabolite and reservoir size (intact and total IPDA values). Targeted quantification was performed for PCS, PAG, PCG, and IAA. Values represent Spearman correlation coefficients between the indicated GDBMs (*x* axis) and metabolites within key pathways (*y* axis). Pathway annotations on the right summarize functional categories significantly associated with GDBM levels. Positive correlations (red) indicate coenrichment between GDBMs and metabolites, while negative correlations (blue) indicate reciprocal relationships suggestive of metabolic suppression. Data were obtained ex vivo from freshly isolated CD4^+^ T cells (*n* = 26 donors). Spearman correlations between HIV reservoir measurements and CD4^+^ T cell metabolites (**A**) are shown with unadjusted *P* values due to the limited number of significant associations, whereas correlations within the metabolomics dataset (**C** and **D**) were corrected for multiple comparisons using the FDR method.

Long-term persistence of the HIV-1 reservoir is tightly linked to CD4^+^ T cell homeostasis, survival, and differentiation (46). While previous studies have implicated immune activation and cellular metabolism in reservoir maintenance (38, 47, 48), direct assessments of how cell-associated metabolic states influence reservoir size remain limited. As our study leveraged untargeted metabolomic profiling of primary CD4^+^ T cells, we sought to determine whether metabolic pathway activity within the CD4^+^ T cell compartment associates with HIV-1 reservoir metrics. In parallel, given the strong linkage between cell-associated GDBMs and CD4^+^ T cell metabolic remodeling, we further examined whether cell-associated GDBM abundance tracks with metabolic pathways implicated in reservoir persistence. To address this, we examined whether the metabolic pathways associated with GDBMs cell level are linked to HIV-1 reservoir persistence, as measured by the intact proviral DNA assay (IPDA). Global correlation analysis between CD4^+^ T cell metabolite abundances and intact HIV-1 reservoir size revealed a bifurcated pattern of associations (Figure 2A). Of the 342 detected cell-associated metabolites, 92 showed significant correlations with intact proviral DNA, segregating into metabolites negatively or positively associated with reservoir size. Figure 2B illustrates the strong positive correlation between intact and total HIV-1 DNA measured in matched CD4^+^ T cell samples, validating the reservoir quantification and enabling comparative metabolic analyses. To resolve pathway-level relationships, we next examined the top metabolites contributing to negative (Figure 2C) or positive (Figure 2D) correlations with intact reservoir size. Metabolites negatively associated with the intact reservoir were enriched in pathways related to nucleotide biosynthesis, amino acid metabolism, and mitochondrial function, whereas metabolites positively associated with the intact reservoir mapped to pathways involved in lipid metabolism, purine and pyrimidine metabolism, and the TCA cycle. Notably, individual GDBMs, including PCS, PAG, PCG, and IAA, did not directly correlate with intact proviral DNA; however, PCS and PAG were positively associated with metabolic pathways that were themselves positively correlated with intact reservoir size, whereas PCG and IAA were linked to pathways exhibiting negative associations (Figure 2, C and D).These divergent patterns suggest that GDBMs collectively influence the immunometabolic landscape of CD4^+^ T cells rather than acting as direct correlates of reservoir burden. Together, these data identify cell-associated GDBMs as previously unrecognized contributors to metabolic programs associated with HIV-1 reservoir persistence and latency in vivo.

### Cell-associated PCS levels are linked to altered marker expression and shifts in CD4^+^ T cell subsets ex vivo.

**Figure 3:**
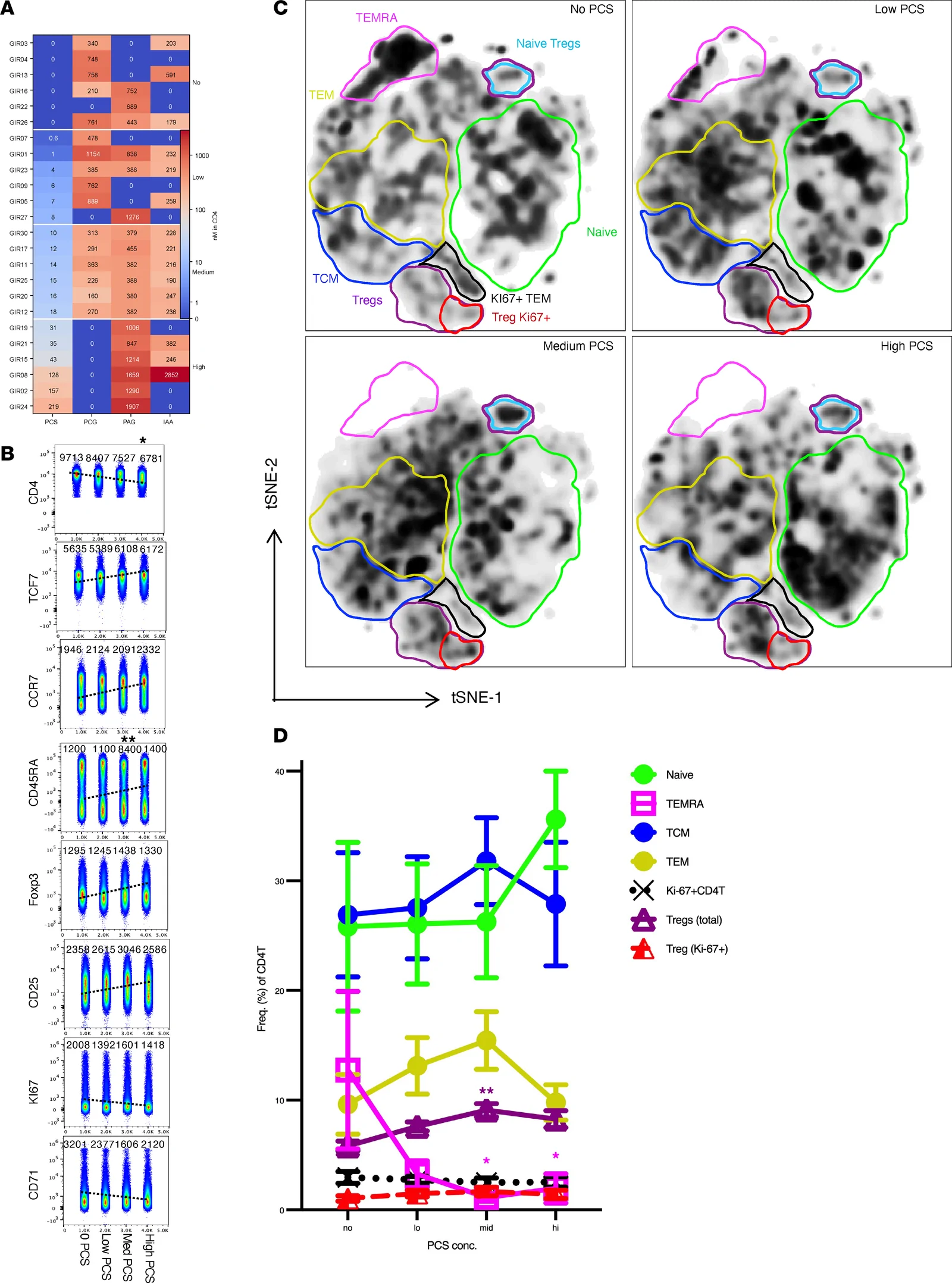
Ex vivo phenotypic characterization of CD4^+^ T cells stratified by cell-associated PCS levels in PLWH. (**A**) Heatmap summarizing cell-associated PCS concentrations in CD4^+^ T cells across individual donors (*n* = 24), grouped into 4 categories based on PCS levels: no PCS, low PCS, medium PCS, and high PCS (6 donors per group). Concentrations (nM) are shown for PCS, PAG, PCG, and IAA in each sample. (**B**) Flow cytometric pseudoplots showing CD4, TCF7, CCR7, CD45RA, FOXP3, CD25, Ki-67, and CD71 expression. Plots represent concatenated files for each PCS group. Median fluorescence intensity (MFI) values for each marker are indicated numerically within individual plots. (**C**) t-SNE plots of CD4^+^ T cells demarked by FlowSOM-defined clusters corresponding to canonical T cell subsets, including naive, TCM, TEM, TEMRA, total Tregs, naive Tregs, and Ki-67^+^ Tregs. Plots represent pooled data from each PCS group. (**D**) Quantification of CD4^+^ T cell subset frequencies as a percentage of total CD4^+^ T cells in each PCS group. Data represent mean ± SD. Statistical significance was assessed using 1-way ANOVA with multiple comparisons correction. *P < 0.05, **P < 0.01.

We then focused our analysis on PCS to determine how cell-associated PCS concentrations shape CD4^+^ T cell biology ex vivo. We stratified peripheral blood samples from PLWH into 4 groups based on cell-associated PCS concentrations measured in CD4^+^ T cells — no PCS, low PCS, medium PCS, and high PCS, with 6 individuals per group as listed in Figure 3A. This stratification enabled us to assess how PCS cell-level correlates with CD4^+^ T cell phenotype and subset composition ex vivo without external stimulation. Using concatenated FlowJo files for each group, we analyzed the median fluorescence intensity (MFI) of key surface and intracellular markers associated with differentiation, homeostasis, proliferation, and regulatory function. A clear reduction in CD4 expression was observed with increasing PCS levels (Figure 3B). Concurrently, there was a progressive increase in the expression of TCF7, CCR7, CD45RA, FOXP3, and CD25, all of which are associated with naive-like, central memory T cell (TCM), or Treg programs. In contrast, markers of proliferation and activation, namely Ki-67 and CD71, exhibited a notable decrease with rising PCS levels, reinforcing the interpretation that high PCS may impair CD4^+^ T cell proliferative capacity. We then applied t-SNE visualization flow self-organizing map (FlowSOM) to assess CD4^+^ T cell landscape changes across PCS concentration groups (Figure 3C and Supplemental Figure 3). With increasing PCS, a visible shift in subset distribution emerged. Specifically, there was a marked reduction in the terminally differentiated effector memory CD45RA (TEMRA) or cytotoxic CD4^+^ T cell population; in contrast, Tregs FOXP3^+^ showed a significant increase in frequency across the PCS gradient, including Ki-67^+^ proliferative Tregs (Figure 3C). Quantitative analysis confirmed these patterns. TEMRA cells significantly declined, while Tregs (total and Ki-67^+^ Tregs) exhibited a statistically significant increase in frequency at higher PCS concentrations (Figure 3D). Although not statistically significant, there was a consistent trend toward increased frequencies of naïve, TCM, and effector memory CD4^+^ T cells (TEM) in the mid and high PCS groups. Additionally, a trend toward decreased Ki-67^+^ TEM cells was observed, further supporting a PCS-associated reduction in CD4^+^ T cell proliferative activity. To assess whether the phenotypic associations observed in PLWH reflected a disease-specific effect or a general property of PCS exposure, we performed the same stratification analysis in CD4^+^ T cells from healthy controls (HC; *n* = 10), stratified into no, medium, and high intracellular PCS groups (Supplemental Figure 4). Several features of the PLWH phenotype were recapitulated in HC; increasing PCS concentrations were associated with declining CD4 MFI, progressive upregulation of TCF7, and induction of FOXP3 and CD25, consistent with a PCS-intrinsic effect on stem-like and regulatory transcriptional programs independent of HIV status. TEMRA frequencies remained low and stable across PCS groups in HC, mirroring the pattern observed in PLWH. CCR7 and CD45RA did not follow the same directional trends as in PLWH, and these differences were not statistically significant in either cohort.

Collectively, although multiple GDBMs were detected intracellularly at comparable concentrations in CD4^+^ T cells from PLWH, the phenotypic shifts described above, TEMRA reduction, Treg induction, and suppression of proliferative capacity, tracked most strongly and consistently with intracellular PCS levels, and they were recapitulated in HCs, establishing PCS as the dominant intracellular determinant of CD4^+^ T cell phenotypic remodeling among codetected GDBMs. The presence of other intracellular GDBMs, including PAG, PCG, and IAA, did not reproduce this coordinated phenotypic program, positioning PCS as a candidate microbiome-derived driver of CD4^+^ T cell immune aging independent of HIV disease status.

Together, these immunophenotypic changes parallel the metabolic alterations described in Figure 1, where higher cell-associated PCS concentrations correlated with disruptions in TCA cycle intermediates and multiple metabolic pathways. The observed reduction in Ki-67 and CD71, coupled with the accumulation of TCF7^+^, CCR7^+^, and FOXP3^+^ cells, may reflect a metabolically constrained environment, likely shaped by PCS-induced mitochondrial dysfunction or impaired bioenergetics. Taken together with the metabolic alterations observed in Figure 1, these data suggest that cell-associated PCS modulates CD4^+^ T cell metabolism and differentiation, promoting a low-proliferative, Treg-skewed phenotype with reduced effector turnover.

### Single-cell transcriptomics reveals that cell-associated PCS skews CD4^+^ T cells toward regulatory, stem-like, and senescence-associated transcriptional states ex vivo.

**Figure 4:**
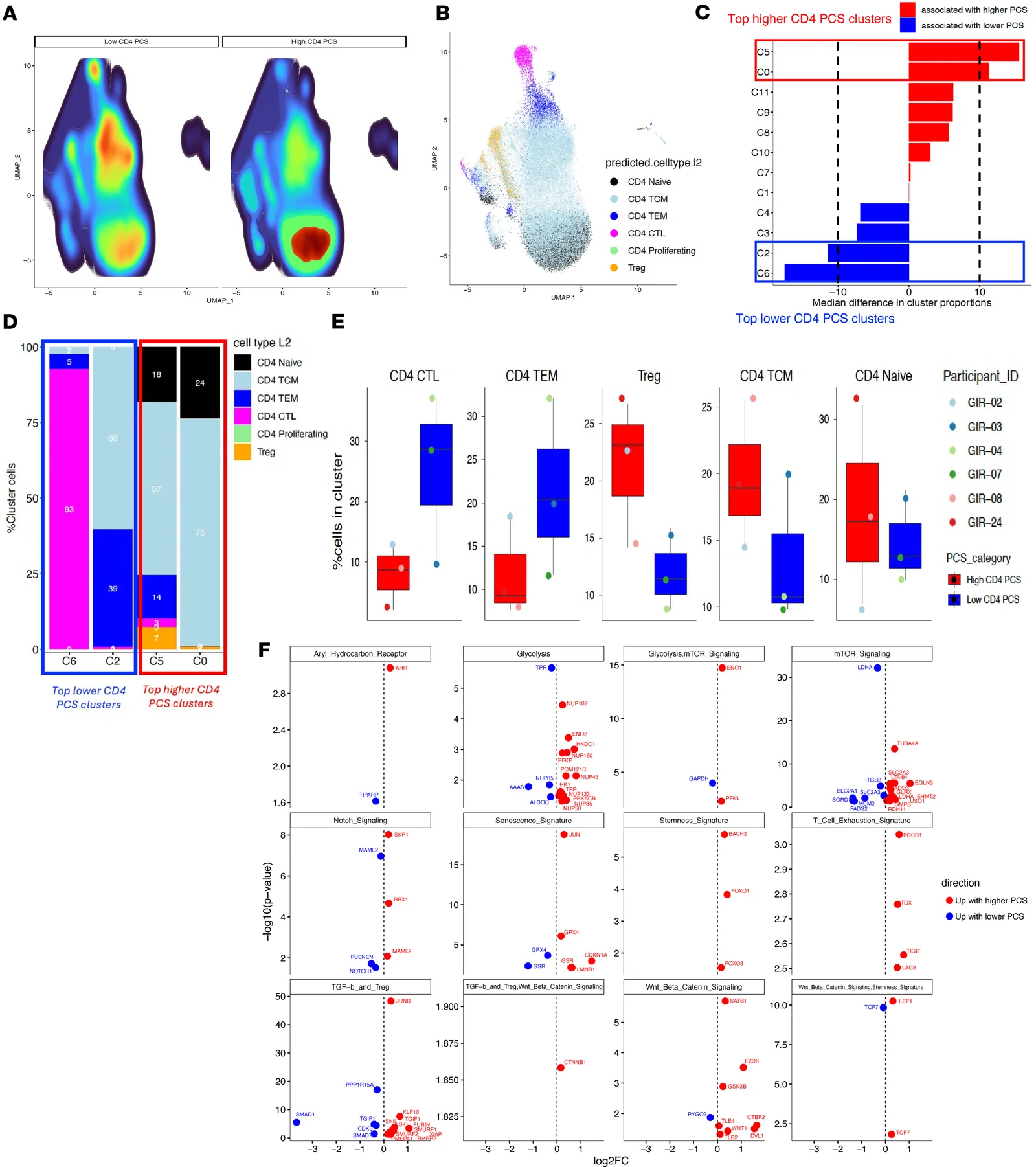
Single-cell transcriptomic analysis of CD4^+^ T cells from participants with high and low cell-associated PCS concentrations. (**A**) UMAP density plots of CD4^+^ T cells from 6 individuals with low (left) or high (right) cell-associated PCS levels. (**B**) UMAP projection of all CD4^+^ T cells colored by predicted CD4^+^ T cell subset identity, annotated by reference mapping to a CITE-seq PBMC multimodal reference. (**C**) Median difference in per-participant cluster proportions between high and low PCS groups; clusters with a median difference exceeding 10% were considered differentially abundant. Red bars indicate clusters enriched in PCS-high donors; blue bars indicate clusters enriched in low PCS donors. (**D**) Stacked bar plot showing predicted CD4^+^ T cell subset identity in clusters C2, C6, C5, and C0 (C5/C0, top high-PCS clusters; C2/C6, top low-PCS clusters). (**E**) Subset percentages within these clusters by individual donor (red, high CD4 PCS; blue, low CD4 PCS). (**F**) Volcano plots of differentially expressed genes within indicated gene signature modules, comparing high versus low PCS clusters. Genes were identified using the Wilcoxon rank-sum test in Seurat (FindMarkers) and summarized by mean log_2_ fold change and mean −log_10_ *P* value across clusters (nominal *P* < 0.05). *n* = 3 donors per group.

To investigate whether cell-associated PCS concentrations shape the transcriptional landscape of CD4^+^ T cells ex vivo, we performed scRNA-seq on sorted CD4^+^ T cells from 6 participants: 3 donors with high PCS (GIR-24, GIR-08, GIR-02) and 3 with low PCS levels (GIR-07, GIR-03, GIR-04). This analysis aimed to validate and expand upon the phenotypic differences previously observed using flow cytometry and FlowSOM clustering. As shown in Figure 4A, UMAP projections revealed clear density differences between low and high PCS samples, suggesting that PCS-associated shifts subpopulation distribution. Clustering analysis of all CD4^+^ T cells identified 12 transcriptionally distinct clusters that were annotated into known CD4^+^ T cell subsets (naive, TCM, TEM, CTL, and proliferating) using established classification algorithms (Figure 4B). Differential abundance analysis (Figure 4C) showed that clusters C5 and C0 were significantly enriched in cells from PCS-high donors, while clusters C2 and C6 were more abundant in low PCS donors. Annotation of the cluster compositions revealed that clusters C5 and C0 were predominantly composed of Tregs and TCM, respectively, whereas C2 and C6 were enriched for TEMRA-like CD4^+^ cytotoxic T lymphocytes (CTL) and TEM cells (Figure 4D). Importantly, these patterns were consistent across individual donors as shown in Figure 4E, underscoring the reproducibility of PCS-associated transcriptional states. To further explore the molecular signatures associated with PCS levels, we performed gene expression comparisons between clusters enriched in high versus low PCS donors. As shown in Figure 4F, CD4^+^ T cells from PCS-high participants upregulated gene modules associated with the AhR pathway, Wnt/B-catenin signaling, TGF-β/Treg differentiation, stemness, and cellular senescence. In AhR pathway, *AHR* expression was significantly enriched in CD4^+^ T cells from PCS-high individuals; within the TGF-β and Treg signature, a distinct upregulation of multiple genes associated with Treg function was observed in PCS-high samples, including *JUNB*, *KLF10*, *SMURF2*, *FURIN*, consistent with transcriptional bias toward Treg polarization. In the Wnt/B-catenin signaling module, PCS-high CD4^+^ T cells showed elevated expression of *SATB1*, *FZD6*, *GSK3B*, *WNT1*, *CTBP2*, and *DVL1*, suggesting active Wnt pathway engagement. The T cell exhaustion signature was also prominently enriched in PCS-high donors, including elevated expression of inhibitory receptors *PDCD1* (*PD-1*), *TOX*, *TIGIT*, and *LAG3*. Consistent with a quiescent or less differentiated state, the stemness signature was enhanced in PCS-high samples with increased expression of *BACH2*, *FOXO1*, *FOXO3*, and *TCF7*, key transcription factors that Treg memory and self-renewal. Finally, the senescence signature revealed strong enrichment of *JUN*, *GPX4*, *GSR*, *CDKN1A* (*p21*), and *LMNB1* in PCS-high CD4^+^ T cells, supporting the hypothesis that PCS drives a senescence-like transcriptional program ex vivo. Together, these data validate our prior flow cytometry findings and demonstrate that cell-associated PCS levels stratify CD4^+^ T cells into distinct transcriptional states. High PCS levels are associated with regulatory and senescence-like profiles, while low PCS levels correlate with effector and cytotoxic phenotypes. Together, these data support a model in which cell-associated PCS influences metabolic remodeling alongside phenotypic and gene expression programs in CD4^+^ T cells ex vivo.

### PCS modulates the transcriptomic and the proteomic profiling of CD4^+^ T cells in vitro.

**Figure 5:**
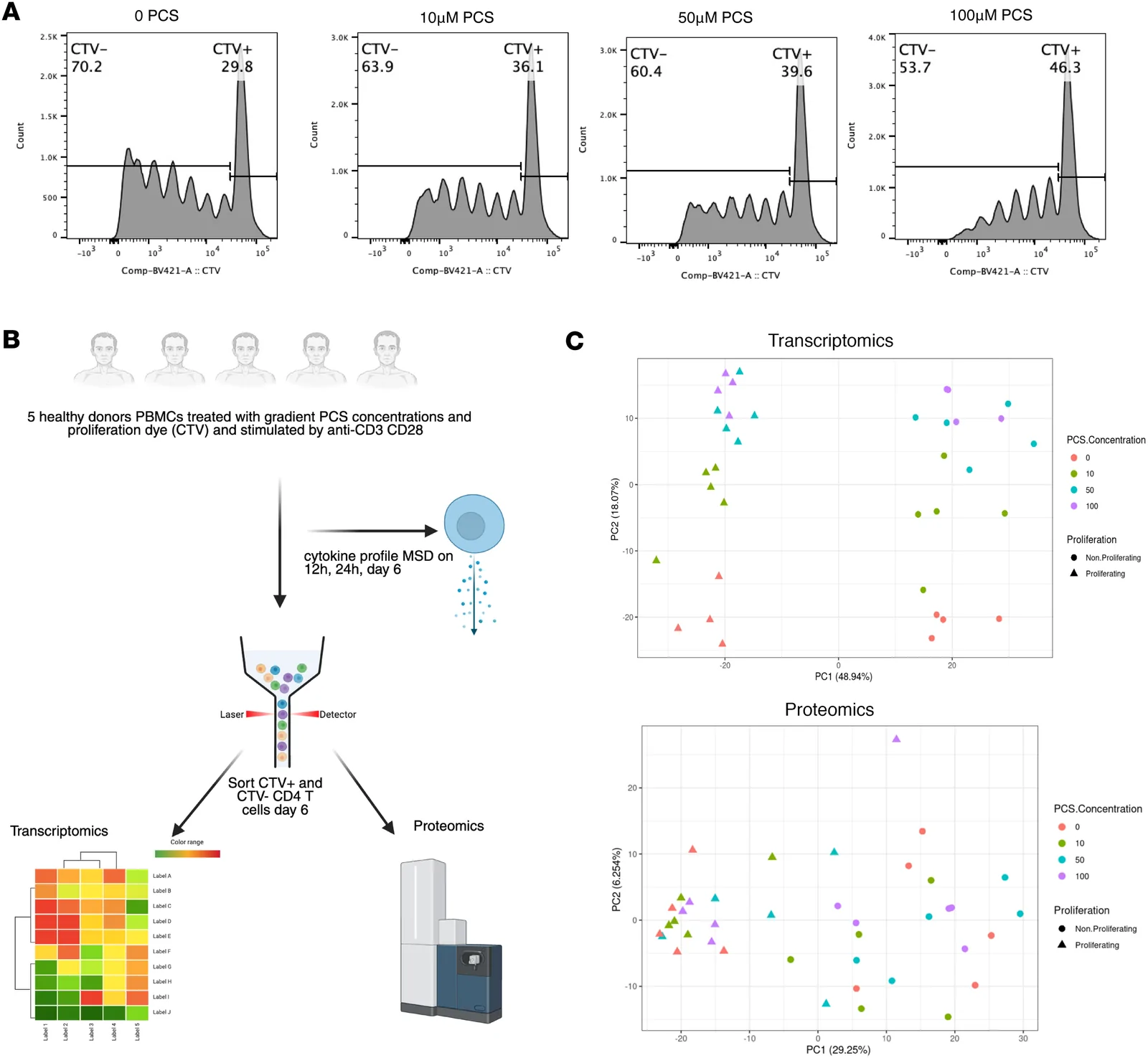
PCS modulates CD4^+^ T cell proliferation and induces coordinated transcriptomic and proteomic remodeling. (**A**) Representative histograms showing proliferation of CD4^+^ T cells labeled with CellTrace Violet (CTV) and stimulated with anti-CD3/CD28 in the presence of increasing concentrations of PCS (0, 10, 50, or 100 μM) for 6 days. CTV^hi^ (nonproliferating) and CTV^lo^ (proliferating) populations are indicated. Representative histograms are shown from *n* = 5 independent donors. (**B**) Schematic of the experimental workflow. PBMCs from 5 healthy donors were labeled with CTV and stimulated with anti-CD3/CD28 in the presence of increasing PCS concentrations. On day 6, CD4^+^ T cells were sorted for CTV^hi^ and CTV^lo^ and subjected to transcriptomic and proteomic analysis. Supernatants were collected for cytokine profiling (MDS), and CD4^+^ T cells were sorted into proliferating and nonproliferating subsets for bulk RNA-seq and proteomic analysis. (**C**) Principal component analysis (PCA) plots of transcriptomic (left) and proteomic (right) datasets from sorted CD4^+^ T cell populations. PCA was performed on variance-stabilized transcriptomic and proteomic data using Seurat. Percentage of variance explained by each principal component is indicated on the respective axes. Data represent *n* = 5 independent donors.

To determine the functional and molecular effect of PCS on CD4^+^ T cells as well as the mechanism of action, we conducted series of in vitro assays. Peripheral blood mononuclear cells (PBMCs) were stimulated with anti-CD3/CD28 in the presence of increasing concentrations of PCS. As shown (Figure 5A), PCS impaired CD4^+^ T cell proliferation in a dose-dependent manner, as evidenced by a progressive increase in the proportion of nonproliferating (CTV^hi^) cells and a corresponding reduction in proliferating (CTV^lo^) cells at 100 μM PCS and as previously assessed (8). The experimental workflow is summarized in Figure 5B; CD4^+^ T cells from 5 healthy donors were treated with 0, 10, 50, or 100 μM PCS, stimulated for 6 days, and sorted into proliferating and nonproliferating subsets for downstream RNA-seq and proteomic analysis. Cytokine profiling was also performed on cell culture supernatants after 12 hours, 24 hours, and day 6 of cell culture. Principal component analysis (PCA) of both transcriptomic and proteomic data revealed clear segregation by PCS concentration and proliferation status, indicating that PCS induces coordinated and dose-dependent molecular remodeling at both transcriptional and protein levels (Figure 5C). Transcriptomic analysis of CTV^hi^ (nonproliferating) and CTV^lo^ (proliferating) CD4^+^ T cells treated with graded concentrations of PCS revealed broadly similar gene expression patterns across both subsets (Supplemental Figure 5). The consistent transcriptional response observed in both populations supports the conclusion that PCS exerts comparable effects irrespective of proliferation status. Accordingly, we focused our in-depth transcriptomic and proteomic analyses on the proliferating CD4^+^ T cell population.

### PCS induces a senescence program in CD4^+^ T cells in vitro.

**Figure 6:**
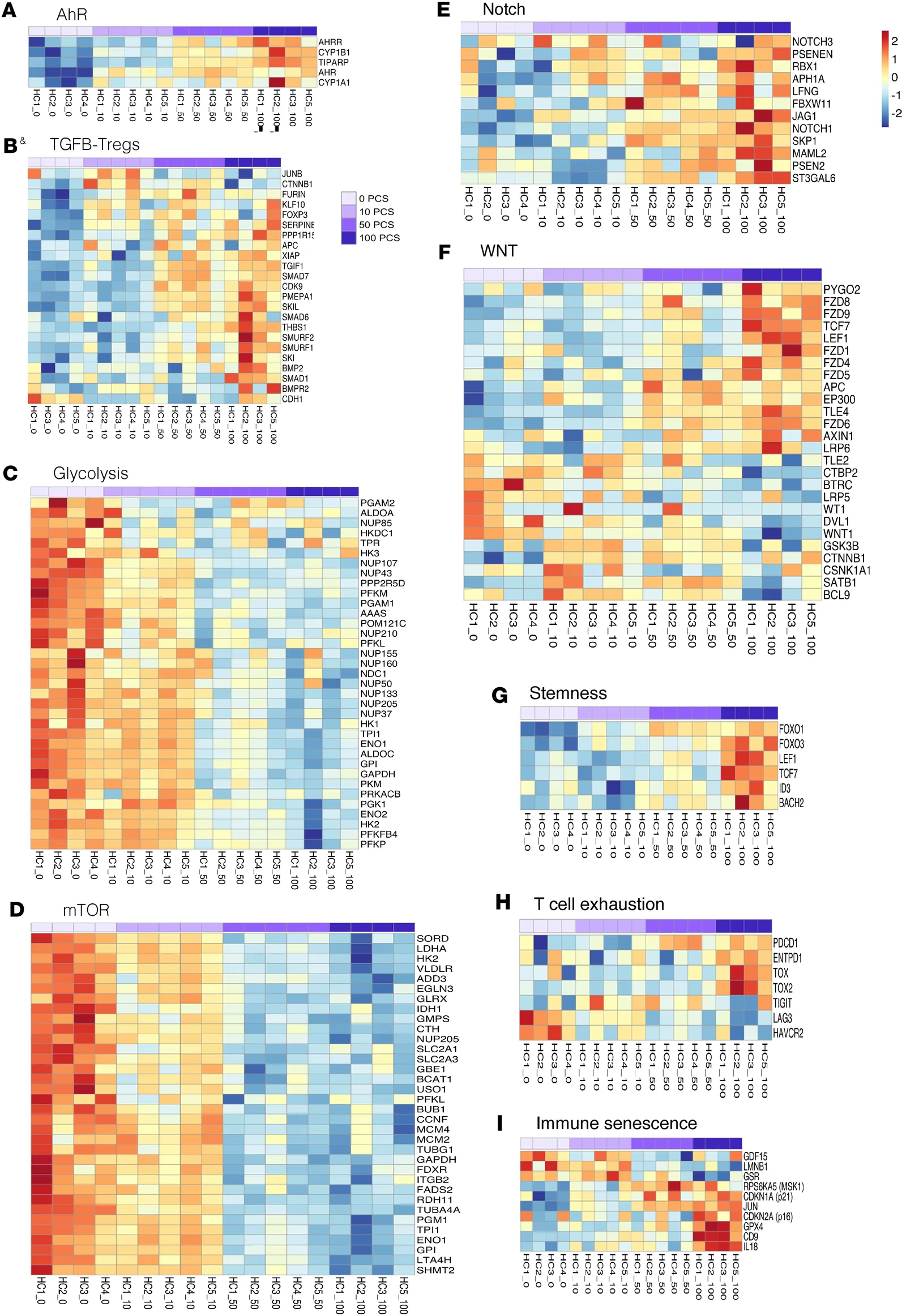
PCS induces transcriptomic reprogramming in proliferating CD4^+^ T cells. Bulk RNA-seq was performed on sorted CTV^lo^CD4^+^ T cells after 6 days of in vitro stimulation with anti-CD3/CD28 in the presence of 0–100 μM PCS. Heatmaps display normalized gene expression (*z* scores) across treatment conditions for selected pathways and gene sets. (**A**) Genes from the AhR signaling pathway. (**B**) TGF-β-Treg signaling pathway. (**C**) Glycolysis pathway. (**D**) mTOR signaling pathway. (**E**) Notch signaling pathway. (**F**) Wnt/B-catenin signaling pathway. (**G**) Stemness-associated genes. (**H**) T cell exhaustion markers. (**I**) Immune senescence markers. Each column represents one sample, and each row corresponds to an individual gene. Expression levels are color-coded from low (blue) to high (red). ^[&]^Indicate that this heatmap was generated from nonproliferating CTV^hi^ cells. Differential gene expression was determined using an FDR-adjusted *P* < 0.05 with a minimum |log_2_ fold change| ≥ 1. *n* = 5 independent donors.

To evaluate the effect of PCS on proliferating CD4^+^ T cells, we performed bulk RNA-seq on sorted CTV^lo^CD4^+^ T cells stimulated in vitro for 6 days with anti-CD3/CD28 in the presence of increasing PCS concentrations (0–100 μM). PCS treatment led to dose-dependent activation of the AhR pathway (*AHR*, *AHRR*, *CYP1B1*, *TIPARP*), suggesting that PCS engages xenobiotic response signaling (Figure 6A) and confirming the ex vivo data obtained by scRNA-seq (Figure 4G). In parallel, TGF-β/Treg signaling pathway (*SMADs*, *FOXP3*) were also upregulated (Figure 6B), consistent with the induction of Treg. PCS exposure also suppressed the expression of key glycolytic genes (*HK1*, *LDHA*, *GAPDH*) (Figure 6C) and mTOR pathway components (*RPS6*, *EIF4EBP1*, *S6K*) (Figure 6D), indicating profound metabolic reprogramming and inhibition of biosynthetic activity. PCS induces Notch signaling pathway (Figure 6E), and the Wnt/B-catenin signaling pathway (Figure 6F), with increased expression of *LEF1*, *TCF7*, and *FZD* family genes. Genes associated with T cell stemness, including *FOXO1*, *FOXO3*, *LEF1*, and *TCF7*, were progressively upregulated with higher PCS concentrations (Figure 6G). Meanwhile, PCS induced prominent expression of T cell exhaustion markers such as *PDCD1*, *TOX*, and *LAG3* (Figure 6H), features typically associated with chronic stimulation and loss of effector function. Finally, genes linked to immune senescence, including *CDKN1A* (*p21*), *CDKN2A* (*p16*), *JUN*, and *CD69*, were markedly upregulated at higher PCS doses (Figure 6I), confirming the acquisition of a senescent-like transcriptional profile.

The extracellular PCS concentration gradient exceeding 3,000-fold relative to intracellular levels is thermodynamically inconsistent with passive diffusion (Supplemental Figure 1), implicating active transporter-mediated cellular entry as the mechanism underlying PCS biological dominance. Consistent with this model, bulk RNA-seq analysis of PCS-exposed primary CD4^+^ T cells identified SLCO4A1 as the sole differentially expressed influx transporter, showing dose-dependent downregulation alongside concurrent suppression of efflux transporters ABCC2, ABCC5, and SLC22A13, and coordinated activation of PGE_2_ signaling, autophagy, and GCN2/ISR pathways (Supplemental Figure 6, A–D). To validate SLCO4A1 downregulation at the protein level, we performed *Z*-stack confocal microscopy on CD4^+^ T cells with and without PCS exposure. In the absence of PCS, SLCO4A1 protein displayed discrete membrane-localized distribution consistent with its function as a plasma membrane transporter. Following PCS exposure, SLCO4A1 signal became diffuse and dissociated from the cell membrane, indicating loss of membrane-resident protein. Quantification by corrected total cell fluorescence (CTCF) confirmed a significant reduction in SLCO4A1 protein abundance upon PCS treatment (*P* = 0.0079, *n* = 5; Supplemental Figure 6, E and F). Together, these findings confirm SLCO4A1 downregulation at both transcriptional and protein levels and establish that intracellular PCS accumulation is governed by transporter-mediated retention rather than systemic exposure; they also confirm that this retention licenses downstream convergence on senescence-associated signaling programs.

Altogether, the transcriptional programs induced by PCS in vitro by bulk RNA-seq closely mirror the ex vivo scRNA-seq signatures from high-PCS donors, indicating that PCS drives a consistent CD4^+^ T cell state across experimental systems. The in vitro experiments conducted to elucidate the mechanism of action of PCS revealed activation of similar transcriptional and phenotypic programs — such as senescence, Treg differentiation, and Wnt/B-catenin signaling — as those identified by scRNA-seq analysis of ex vivo CD4^+^ T cells from PCS-high participants. These transcriptional changes reveal that PCS orchestrates a coordinated program in CD4^+^ T cells characterized by xenobiotic sensing, metabolic remodeling and inhibition, stem-like adaptation, immune exhaustion, and convergence toward a senescence-like state.

**Figure 7:**
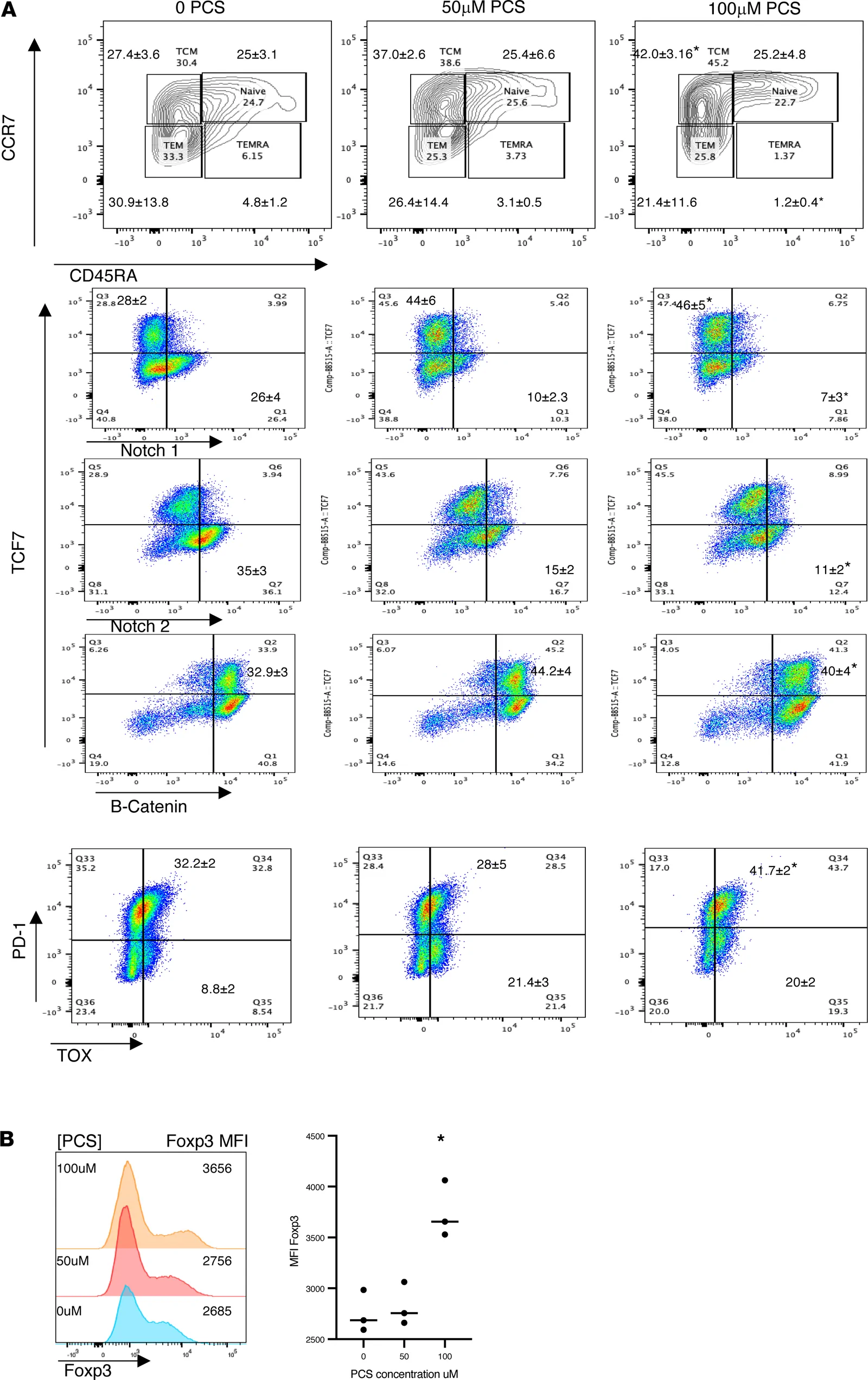
PCS induces senescence markers and alters CD4+ T cell phenotype in vitro. (**A**) Representative flow cytometry plots of CD4+ T cells stimulated with anti-CD3/CD28 for 6 days in the presence of 0, 50, or 100 μM PCS, showing CCR7 versus CD45RA (top panels) and TCF7, Notch1, Notch2, B-catenin, and PD-1 versus TOX (middle and bottom panels). Values in quadrants indicate median ± SD across 3 biological replicates. (**B**) Overlay histogram (left) and quantification (right) of FOXP3 expression in naive CD4+ T cells cultured in the presence of IL-2 and increasing concentrations of PCS (0, 50, or 100 μM) for 5 days. Data in B represent *n* = 3 independent donors. Statistical comparisons were performed using 1-way ANOVA with multiple comparisons correction. *P < 0.05.

**Figure 8:**
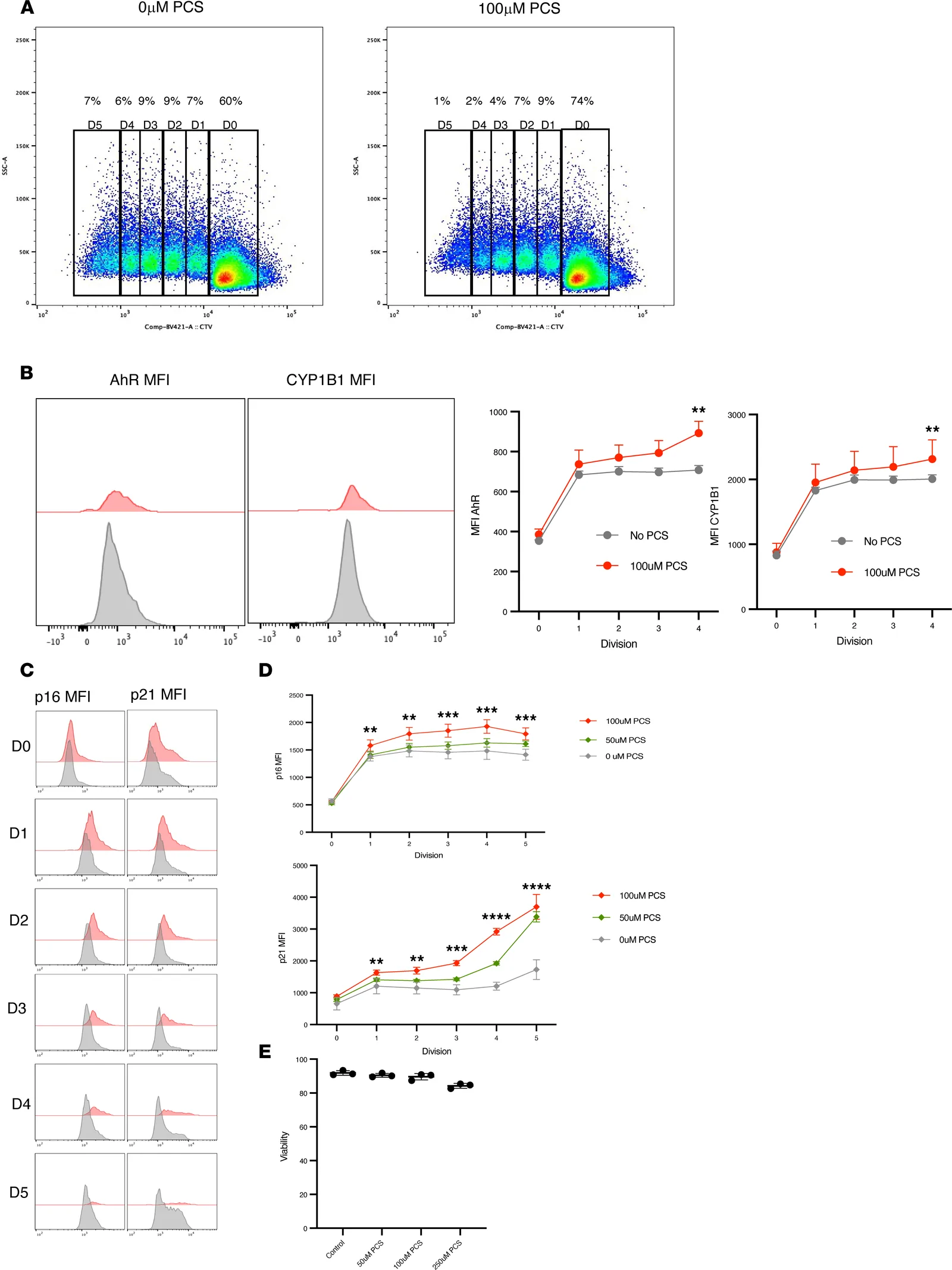
PCS induces an AhR-associated senescence phenotype in CD4^+^ T cells. (**A**) CellTrace Violet dilution plots showing proliferative divisions of CD4+ T cells cultured with or without 100 μM PCS. (**B**) MFI Flow cytometry histograms (left) and quantification (right) of AhR and CYP1B1 expression across divisions (Div0–Div4) for each PCS condition. (**C**) Flow cytometry histograms of p16 and p21 expression across divisions (Div0–Div5) for each PCS concentration. (**D**) Quantification of p16 and p21 MFI across cell divisions in each PCS condition. (**E**) Viability of CD4+ T cells after 6 days of culture with or without PCS, assessed by fixable viability dye. Data represent *n* = 3 independent donors. Statistical comparisons were performed using 1-way ANOVA with multiple comparisons correction: **P < 0.01; ***P < 0.001; ****P < 0.0001.

To investigate the phenotypic effect of PCS exposure during CD4^+^ T cell activation and to validate the transcriptomic signature, we cultured human CD4^+^ T cells in vitro with anti-CD3/CD28 in the presence of increasing concentrations of PCS (0, 50, 100 μM). Flow cytometry analysis of CCR7 versus CD45RA (top panels; Figure 7A) showed a progressive redistribution of T cell subsets. Specifically, the frequency of CCR7^+^CD45RA^–^ TCM cells increased from 27.4.3% ± 3.6% at 0 μM PCS to 37.0% ± 2.6% at 50 μM and 42.0% ± 3.16% at 100 μM. Meanwhile, TEMRA (CCR7^–^CD45RA^+^) cells decreased from 4.8% ± 1.2% (0 μM) to 3.1% ± 0.5% (50 μM) and 1.2% ± 0.4% (100 μM), consistent with the PCS-associated TEMRA contraction observed ex vivo (Figure 3D). TEM cells declined (30.9% ± 13.8%, 26.4% ± 14.4%, 21.4% ± 11.6%), while naive cell frequencies remained relatively stable. In the middle and lower panels, PCS promoted a strong dose-dependent induction of TCF7 (28% ± 2%, 44% ± 6%, 46% ± 5%) and B-catenin (32.9% ± 3%, 44.2% ± 4%, 40.0% ± 4%) expression, supporting the activation of the Wnt signaling pathway. Additionally, Notch1 and Notch2 surface expression was reduced (Notch1^+^: 26% ± 4%, 10% ± 2.3%, 7% ± 3%; Notch2^+^: 35.3% ± 3%, 15% ± 2%, 11% ± 2%), indicating ligand engagement and downstream signaling. These phenotypic changes parallel the transcriptional activation of the Wnt/B-catenin and Notch pathways identified in RNA-seq analysis (Figure 6, D–F) and altogether reflect a PCS-driven shift toward a regulatory or noneffector CD4^+^ T cell state. In vitro assays demonstrated that PCS directly upregulates FOXP3 protein expression (Figure 7B). Stimulation of naive CD4^+^ T cells with anti-CD3/CD28 in the presence of PCS led to a clear dose-dependent induction of FOXP3^+^ cells. These data validate the Treg induction detected in the ex vivo flow cytometric (Figure 3, C and D) and in the scRNA-seq (Figure 4, F and G) analyses. To validate AhR pathway activation suggested by the transcriptomic analysis (Figure 4G and Figure 6A), we next assessed AhR protein expression and its canonical downstream target CYP1B1 in CD4^+^ T cells cultured with or without PCS. Flow cytometry analysis across proliferative divisions (D0 to D4) (Figure 8A) revealed a progressive increase in both AhR and CYP1B1 MFI with increasing PCS concentrations (Figure 8B). Cells exposed to 100 μM PCS displayed a significant elevation in AhR and CYP1B1 expression beginning at the first division and persisting through later divisions, compared with untreated controls (*P* < 0.01). These findings confirm that PCS activates the AhR signaling pathway in proliferating CD4^+^ T cells, consistent with the observed upregulation of AhR-related genes (including CYP1B1 and AhR) in transcriptomic datasets. To assess senescence-related responses, we quantified p16 and p21 expression across cell divisions, tracked by CTV dilution (Figure 8, A and C). PCS markedly increased p16 and p21 expression in proliferating CD4^+^ T cells, with the highest MFI detected in late divisions. Quantitative analysis confirmed a significant, dose-dependent elevation of both markers in PCS-treated cells across all divisions (Figure 8, C and D). Importantly, PCS treatment did not compromise cell viability (Figure 8E), indicating that reduced proliferation was not attributable to cytotoxicity.

The aggregate findings show that PCS reprograms CD4^+^ T cell differentiation, metabolic remodeling, and signaling by activating Wnt/B-catenin and Notch pathways, enhancing FOXP3 expression, and inducing senescence-associated proteins. These changes integrate with transcriptomic evidence (Figure 4 and Figure 6) to support a model in which PCS imposes a nonproliferative, regulatory-like, and senescent transcriptional state that may contribute to long-term T cell dysfunction and immune cell aging.

PCS exposure induced a coordinated proteomic shift from immune effector programs toward mitochondrial and stress-adaptive states, characterized by downregulation of proteins involved in TCR signaling, cytotoxicity, and zinc homeostasis and by upregulation of mitochondrial quality control, NAD^+^ biosynthesis, and transcriptional stress response proteins (Supplemental Figure 7). Consistent with these findings, PCS suppressed proinflammatory and lineage-defining cytokine production and blunted both Th1 and Th2 polarization, as evidenced by reduced T-bet and GATA3 expression in PCS-exposed CD4^+^ T cells (Supplemental Figure 8).

Altogether, our comprehensive multi-omics analysis reveals that PCS exerts profound and coordinated effects on CD4^+^ T cell biology. Ex vivo and in vitro transcriptomic profiling of CD4^+^ T cells exposed to PCS uncovered a shift toward immunoregulatory and stem-like states, with activation of TGF-β, Wnt/B-catenin, and AhR signaling pathways, alongside suppression of mTOR, and glycolysis. These transcriptional changes were mirrored at the proteomic level by downregulation of proteins essential for T cell signaling, cytotoxicity, and antigen trafficking and by upregulation of proteins involved in mitochondrial maintenance, metabolic stress adaptation, and transcriptional repression. Functionally, PCS impaired CD4^+^ T cell proliferation, suppressed proinflammatory and lineage-defining cytokines, and blunted Th1 and Th2 polarization, as shown by reduced expression of the transcription factors T-bet and GATA3 (Supplemental Figure 8). Together, these findings suggest that PCS reprograms CD4^+^ T cells into a metabolically restrained and functionally suppressed state, characterized by induction of senescence program that leads to CD4^+^ T cell immune cell aging characteristics of immune cells in PLWH.

## Discussion

**Figure 9:**
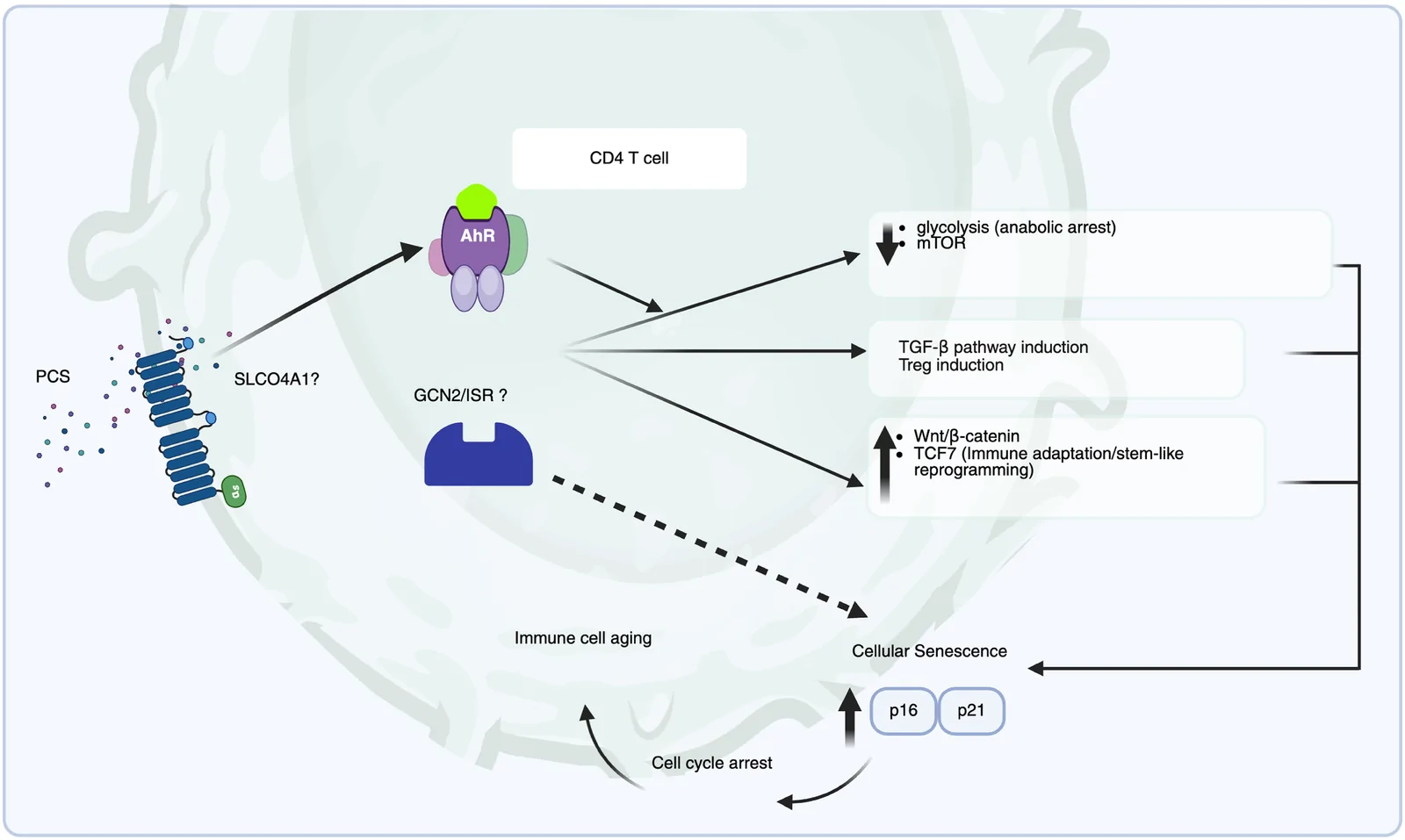
Proposed mechanistic model of PCS-driven CD4^+^ T cell reprogramming and immune aging. PCS enters CD4^+^ T cells via SLCO4A1-mediated active transport. Once intracellular, PCS engages 2 parallel signaling axes. First, PCS acts as an aryl hydrocarbon receptor (AhR) agonist, suppressing glycolytic and mTOR-dependent anabolic programs, inducing TGF-β pathway activation and Treg programming, and upregulating Wnt/B-catenin signaling and TCF7-driven stem-like transcriptional reprogramming. Second, PCS activates the GCN2/integrated stress response (ISR) axis, potentially through direct amino acid sensing operating in parallel with or downstream of AhR activation, further amplifying the senescence program. Convergence of these 2 pathways drives upregulation of the canonical senescence markers p16 and p21, leading to cell cycle arrest and establishment of a stable cellular senescence state. Together, these interconnected programs establish a self-reinforcing immune aging phenotype characterized by metabolic arrest, Treg induction, stem-like transcriptional reprogramming, and cellular senescence, mirroring the premature immune aging observed in PLWH on long-term ART. Solid arrows indicate supported mechanistic connections; dashed arrow indicates a proposed but not yet experimentally confirmed link between GCN2/ISR activation and senescence in this context. Question marks denote pathways requiring direct experimental validation.

In this study, we show that GDBMs remodel metabolic pathways in human CD4^+^ T cells and reshape key aspects of their cellular biology. Integrating ex vivo metabolomics with scRNA-seq, bulk RNA-seq, and high-dimensional cytometry (t-SNE/FlowSOM), we identify a coherent program of metabolic remodeling, senescence induction, and diminished effector potential driven by cell-associated PCS, positioning this GDBM as a key cell-intrinsic regulator of CD4^+^ T cell aging. The present study establishes intracellular PCS accumulation as the dominant determinant of CD4^+^ T cell senescence, transcriptional reprogramming, and metabolic dysfunction in PLWH, yet the mechanism by which PCS drives these changes warrants consideration. The extracellular PCS concentration gradient exceeding 3,000-fold relative to intracellular levels is thermodynamically inconsistent with passive diffusion, implicating active transporter-mediated cellular entry. SLCO4A1, a member of the organic anion transporting polypeptide (OATP) family that mediates uptake of sulfated organic anions, thyroid hormones (T3, T4), and prostaglandins (PGE_2_) (49–51), was identified by bulk RNA-seq as the sole differentially expressed influx transporter, showing dose-dependent downregulation confirmed at the protein level by confocal microscopy (Supplemental Figure 6, E and F). This pattern is consistent with a feed-forward retention loop in which PCS entry suppresses its own transporter, progressively trapping PCS intracellularly. Concurrent suppression of efflux transporters ABCC2, ABCC5 (52), SLC22A1 (53), and SLC22A13 (54) further amplifies intracellular retention (Supplemental Figure 6A). Beyond transporter dysregulation, intracellular PCS accumulation was associated with coordinated transcriptional reprogramming across 4 convergent pathways: dual PGE_2_ overproduction and impaired clearance via PTGS2 induction (55–57); HPGD suppression (56); autophagy engagement through ATG5, BECN1, and MAP1LC3B induction; and GCN2/ISR axis activation (58–60) evidenced by upregulation of ATF3 (61) and ATF4 (62), all dose-dependent and directionally consistent across independent donors (Supplemental Figure 6, B–D). Based on the totality of evidence, we propose a mechanistic model in which PCS uptake into CD4^+^ T cells involves SLCO4A1-mediated active transport and engages 2 parallel intracellular signaling axes (Figure 9). First, intracellular PCS acts as an AhR agonist, suppressing glycolysis and mTOR-dependent anabolic signaling, inducing TGF-β pathway activation and Treg programming, and upregulating Wnt/B-catenin and TCF7-driven stem-like reprogramming, collectively converging on cellular senescence marked by p16 and p21 induction. Second, PCS engages the GCN2/ISR axis through a mechanism that may operate in parallel with or downstream of AhR activation — potentially reflecting direct amino acid sensing and thereby amplifying the senescence program and contributing to immune cell aging. Together, these convergent pathways establish a self-reinforcing senescence state characterized by metabolic arrest, Treg induction, and stem-like transcriptional reprogramming that mirrors the immune aging phenotype observed in PLWH on long-term ART. The full mechanistic framework is comprehensively delineated in our recent review (63).

Our findings extend the remote sensing and signaling theory (RSST) into the immune compartment. RSST posits that multispecific organic anion transporter (OAT), organic anion-transporting polypeptide (MRP), and multidrug resistance-associated protein (MRP) transporters function as a remote sensing and signaling network coordinating interorgan and host-microbiome communication through transporter-mediated uptake and retention of circulating metabolites (64–68). Consistent with this framework, GDBMs are actively transported into gut epithelial cells, hepatocytes, and renal proximal tubular cells, where they engage intracellular signaling cascades, most notably AhR activation, that regulate inflammatory tone, barrier integrity, and metabolic homeostasis in a tissue-specific manner (64–68). While RSST has been primarily characterized in these epithelial and parenchymal compartments, our data suggest that the same transporter-mediated sensing and signaling principle operates within circulating CD4^+^ T cells. Specifically, SLCO4A1-mediated PCS uptake combined with concurrent suppression of efflux transporters ABCC2, ABCC5, SLC22A1, and SLC22A13 creates an intracellular retention state that triggers convergent downstream signaling across the AhR, GCN2/ISR, and PGE_2_ axes, precisely the type of transporter-orchestrated intracellular signaling that RSST predicts. We propose that PCS-driven transporter dysregulation in CD4^+^ T cells represents a special case of RSST operating within the immune system, in which GDBM hijack the transporter network to enforce intracellular retention, chronic AhR activation, and immune dysfunction. To our knowledge, our findings provide the first evidence of RSST-consistent transporter-mediated sensing and signaling in a circulating immune cell and link this framework to HIV reservoir persistence and immune aging. Whether the RSST-consistent transporter-mediated GDBM sensing and signaling paradigm proposed here for CD4^+^ T cells extends to other immune cell populations remains an important open question that warrants dedicated investigation. The transporter/AhR-senescence axis we describe in CD4^+^ T cells is consistent with evidence from other cell types; indoxyl sulfate, a gut-derived uremic toxin, induces p21-mediated senescence in renal proximal tubular cells via OAT1/OAT3-mediated uptake (69, 70) and activates AhR in macrophages and endothelial cells to drive vascular dysfunction (71, 72). Our findings extend this conserved transporter/AhR-senescence axis to CD4^+^ T cells and uniquely link it to HIV to immune aging and HIV-1 reservoir persistence.

Our observation that PCS induces p16 and p21 in CD4^+^ T cells aligns with growing evidence that T cells undergo senescence in both aging and chronic disease, with p16 and p21 serving as hallmarks of aged T cells associated with reduced proliferative capacity, altered metabolic programming, and increased susceptibility to functional exhaustion (18, 20, 21). These findings position PCS as an upstream inducer of immunosenescence that may exacerbate aging-associated immune decline in PLWH (22, 28, 73). AhR is broadly expressed in immune cells and integrates signals from dietary, microbial, and endogenous ligands to regulate differentiation, metabolism, and immune function, with chronic engagement predisposing lymphocytes to aging-like phenotypes (74–76). Our observation that PCS robustly induces AhR and downstream signaling alongside p16, p21, and metabolic dysfunction positions AhR not merely as a xenobiotic sensor but as a microbiome-responsive regulator of CD4^+^ T cell longevity and fate.

Long-lived and transcriptionally inert CD4^+^ T cell subsets are recognized as critical components of the latent HIV-1 reservoir that persists despite prolonged ART. T memory stem cells harbor the highest per-cell HIV-1 DNA levels and maintain reservoir stability over nearly a decade of ART (77), while senescence-like programs in central memory CD4^+^ T cells have been positively correlated with inducible HIV reservoirs (78). Foundational studies further established that HIV preferentially resides in long-lived memory T cells (46, 79). Together, these findings suggest a unifying model in which HIV-1 capitalizes on both stemness and senescence within the CD4^+^ T cell compartment, phenotypes that confer resistance to cytolytic mechanisms, immune surveillance, and apoptosis. Our data corroborate these findings, demonstrating that PCS induces CD4^+^ T cell senescence characterized by increased expression of p16, p21, and exhaustion markers, a cell state permissive for HIV-1 reservoir maintenance.

The accumulation of metabolic intermediates within CD4^+^ T cells is a hallmark of disrupted pathway activity, as evidenced by canonical examples such as succinate-driven HIF-1α stabilization in macrophages and TCA intermediate accumulation altering redox balance and epigenetic regulation under mitochondrial stress (17, 80–82). The ex vivo correlation network revealed that multiple GDBMs converge on shared metabolic pathways within CD4^+^ T cells, defining a common immunometabolic signature associated with immune dysfunction (Figure 1 and Figure 2). PAG and PCG have not previously been shown to modulate immune cells; here we detect both metabolites at the cellular level in human CD4^+^ T cells. PAG exhibited significant positive correlations with multiple metabolic pathways (Figure 1D) and with pathways positively associated with intact HIV-1 reservoir size (Figure 2D), a pattern similarly observed for PCG. Further studies are needed to address the mechanism of action of PAG and PCG on CD4^+^ T cells. Our data provide evidence that intact and total HIV-1 reservoirs only partially overlap in their metabolic associations, suggesting distinct metabolic dependencies for reservoir maintenance (Figure 2, C and D). The intact reservoir displayed broader associations with dysfunctional central carbon and nucleotide metabolism, indicating that metabolite-driven immune cell dysfunction may preferentially support the persistence of intact, replication-competent proviruses. In contrast, total reservoir size was associated with a narrower set of metabolic pathways. To our knowledge, this is the first study to perform CD4^+^ T cell metabolic profiling demonstrating that intact and total HIV-1 proviruses are associated with distinct metabolic signatures, indicating that these 2 reservoir compartments may be shaped and maintained through different immunometabolic states.

While our findings demonstrate that cell-associated PCS is linked to extensive metabolic, transcriptional, and phenotypic remodeling of CD4^+^ T cells, several considerations are important for contextualizing these observations. Because our analyses are based on cross-sectional ex vivo profiling, they do not establish the temporal sequence or causality of PCS exposure and CD4^+^ T cell reprogramming in vivo. Cell-associated PCS levels likely reflect a dynamic balance of uptake, binding, and clearance that was not longitudinally assessed, potentially underestimating transient or fluctuating intracellular exposures. More broadly, GDBMs remain insufficiently explored in immunity, and future studies will be required to define how their cell-associated accumulation influences diverse immune compartments across disease contexts. Finally, the observed associations between cell-associated GDBMs and HIV-1 reservoir size require direct in vitro validation, which is the focus of ongoing studies in our group.

Our findings support a systems-level model in which GDBMs act as endocrine-like regulators of immune metabolism, influencing the balance between immune activation, quiescence, and senescence. Within this framework, PCS emerges as a prototype effector that recapitulates the broader metabolic dysfunction observed ex vivo, providing a mechanistic entry point to dissect how chronic microbial exposure drives immune aging and shapes T cell homeostasis. Strategies aimed at modulating GDBM levels via gut microbiome targeting or dietary interventions may restore metabolic balance, reverse immune senescence-associated aging, and disrupt HIV reservoir stability.

## Methods

Supplemental Methods are available online with this article.

### Sex as a biological variable.

This study enrolled both male and female participants across all experimental cohorts. In the Immune responders HIV cohort used for primary analyses (Figure 3), participants included both sexes with a predominance of female donors, reflecting the demographics of the HIV^+^ population enrolled at our clinical site. Sex was not designated as a primary independent variable in the current study design, as the central focus was on intracellular GDBM accumulation and its immunometabolic consequences in CD4^+^ T cells. However, both male and female samples contributed to all major analyses including metabolomics, flow cytometry, scRNA-seq, and in vitro functional assays. The findings reported here, including SLCO4A1-mediated intracellular PCS retention, AhR activation, and CD4^+^ T cell senescence and stemness reprogramming, are expected to be relevant to individuals of both sexes, as the underlying mechanisms involve conserved transporter biology and transcriptional regulatory pathways that are not known to be sex-restricted. Future studies with adequate statistical power to examine sex as a biological variable are warranted.

### Statistics.

Statistical analyses were performed using GraphPad Prism (version 11.0, GraphPad Software, San Diego, CA, USA) and R (version 4.5.1, R Core Team, Vienna, Austria). Spearman rank correlation was used to assess associations between continuous variables, including cell-associated and plasma metabolite concentrations, metabolic pathway enrichment scores, and HIV-1 reservoir measurements. For multiple comparisons within the metabolomics dataset, *P* values were adjusted using the FDR method; an FDR-adjusted *P* value less than 0.05 was considered statistically significant. For correlations with HIV reservoir measurements, unadjusted *P* values are reported due to the limited number of significant associations. Differences in CD4^+^ T cell subset frequencies across PCS concentration groups and flow cytometry marker expression across PCS treatment conditions in vitro were assessed using 1-way ANOVA with Tukey’s multiple comparisons correction. A *P* value less than 0.05 was considered statistically significant for all analyses unless otherwise specified.

For RNA-seq and proteomics, differential features were identified at FDR-adjusted *P* < 0.05 and |log_2_ fold change| ≥ 1. For metabolomics, FDR-adjusted *P* < 0.05 was considered significant. Thresholds are indicated in corresponding figure legends. scRNA-seq processing and clustering. CD4^+^ T cell single-cell gene expression data (10x Genomics) from 6 donors stratified into high (*n* = 3) and low (*n* = 3) CD4 PCS groups were analyzed in Seurat. Counts were log-normalized (scale factor 10,000), and the 2,000 most variable genes were identified by variance-stabilizing transformation. Data were scaled and reduced by PCA, and cells were clustered on the first 30 principal components using a shared nearest-neighbor graph (k = 30) followed by Louvain community detection (resolution = 0.6), with the optimal resolution guided by clustree. Batch effects across donors were corrected with Harmony (grouping variable: participant), and Harmony embeddings were used for neighbor finding, clustering, and UMAP visualization. Cell identities were annotated by reference mapping to a CITE-seq PBMC multimodal reference (CD4 T compartment) using SCTransform normalization and supervised PCA anchor transfer; cells with a prediction score ≥ 0.4 were retained. Differential cluster abundance. For each cluster, the proportion of cells contributed by each participant was calculated, and clusters were ranked by the difference in median per-participant proportions between the high and low CD4 PCS groups. Clusters showing a median between-group difference exceeding 10% were considered differentially abundant and selected for downstream analysis. Within each selected cluster, differentially expressed genes between high and low CD4 PCS donors were identified using the Wilcoxon rank-sum test implemented in Seurat’s FindMarkers function (logfc.threshold = 0, min.pct = 0.01). Genes were summarized by their mean log_2_ fold change and mean −log_10_ *P* value across clusters and displayed as volcano plots, with significance assessed at a nominal *P* < 0.05. Mitochondrial, ribosomal, and uncharacterized transcripts were excluded.

### Study approval.

Fifty virally suppressed PLWH on ART were recruited from the Emory CFAR HIV Disease Registry at the Ponce Center, Grady Health System. The cohort had a median age of 59 years (range 40–70), median CD4^+^ T cell count of 735 cells/μL (range 478–1,453), and median ART duration of 10.15 years (range 0.2–25.23). Twenty-four participants underwent untargeted and targeted metabolomic analyses. All participants provided written informed consent under a protocol approved in accordance with the Declaration of Helsinki. Patient characteristics are in Supplemental Table 1.

### Data availability.

Raw and processed scRNA-seq data are deposited in GEO under accession no. GSE328611. Bulk RNA-seq data are deposited in GEO under accession no. GSE329693. Proteomics data are deposited in ProteomeXchange/PRIDE under accession no. PXD077610. Metabolomics data are deposited in the Metabolomics Workbench (Study ID ST004747; DOI: dx.doi.org/10.21228/M86V8D). Supporting data values for all applicable figure panels, including values for all data points shown in graphs and values behind any reported means, are provided in the accompanying Supporting Data Values Excel file. Human subject clinical data are not publicly available due to participant privacy protections but deidentified data may be made available from the corresponding author upon reasonable request. Analysis code is available from the corresponding author upon request.

### Declaration of generative AI and AI-assisted technologies in the writing process.

During the preparation of this work, the authors used ChatGPT and Claude (Anthropic) to improve the language and readability of the manuscript. After using this tool/service, the authors reviewed and edited the content as needed and take full responsibility for the content of the published article. The Introduction paragraphs have been passed through Chatgpt (Version 4). The Discussion paragraphs have been passed through Claude (Sonnet 3.7) from January – March, 2026.

## Author contributions

SAY conceived and wrote the manuscript. ACDS, LF, JW, ALR, SK, SW, LPDB, DEG, and JAT performed experiments. ACS and SK performed scRNA-seq, AT analyzed scRNA-seq, and JAT performed bulk RNAseq with ACDS, DEG, and SW performed proteomics. VCM enrolled participants and obtained samples. JW, DPJ, and LF performed and analyzed metabolomics. AG performed confocal experiments.

## Conflict of interest

VCM has received investigator-initiated research grants (to the institution) and research support from Eli Lilly, Bayer, Gilead Sciences, Merck, and ViiV.

## Funding support

This work is the result of NIH funding, in whole or in part, and is subject to the NIH Public Access Policy. Through acceptance of this federal funding, the NIH has been given a right to make the work publicly available in PubMed Central.

- National Institute on Aging, R01AG076373 (SAY).
- National Institute of Allergy and Infectious Diseases/Emory Center for AIDS Research, P30AI050409 (VCM).
- National Institute of Diabetes and Digestive and Kidney Diseases, U2C-DK119886 (metabolomics data deposition).
- NIH Office of the Director/Common Fund Data Ecosystem, OT2-OD030544 (metabolomics data deposition).

## Supplementary Material

Supplemental data

Supporting data values

## Version 1 07/21/2026

In-Press Preview

## Version 2 08/24/2026

Electronic publication
