# Supplementary material for: Microbiome-derived metabolites shape CD4+ T cell differentiation and immune aging in HIV-1 infection
Source: JCI Insight. 2026 Jul 21;11(16):e204383. doi: 10.1172/jci.insight.204383 (PMC13502178; doi:10.1172/jci.insight.204383)
Supplement: Supplementary file 1 [file jciinsight-11-204383-s270.pdf]

Supplementary Figure 1

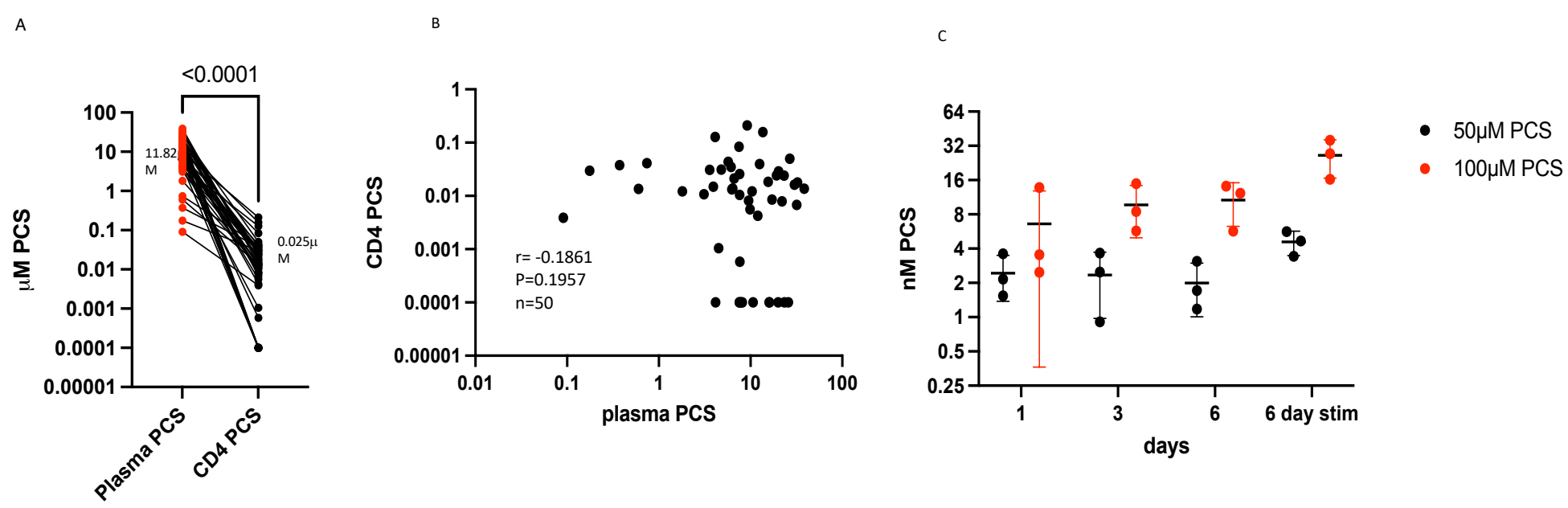

Supplementary Figure 1. PCS concentrations in plasma and CD4+ T-cells from PLWH reveal tissue-specific accumulation and lack of correlation. (**A**) Mass spectrometry was used to quantify PCS concentrations in paired plasma and CD4+ T-cell samples from 50 PLWH. The mean PCS concentration in plasma was 11.82 µM, whereas the mean cell-associated concentration in CD4+ T-cells was 0.025 µM. **(B)** Spearman correlation analysis between plasma and CD4+ T-cell PCS concentrations. **(C)** Cell-associated PCS accumulation in CD4+ T-cells is shown from a separate experiment in which cells were incubated in vitro with 50 or 100 µM PCS for 1, 3, or 6 days, with or without TCR stimulation.

Supplementary Figure 2

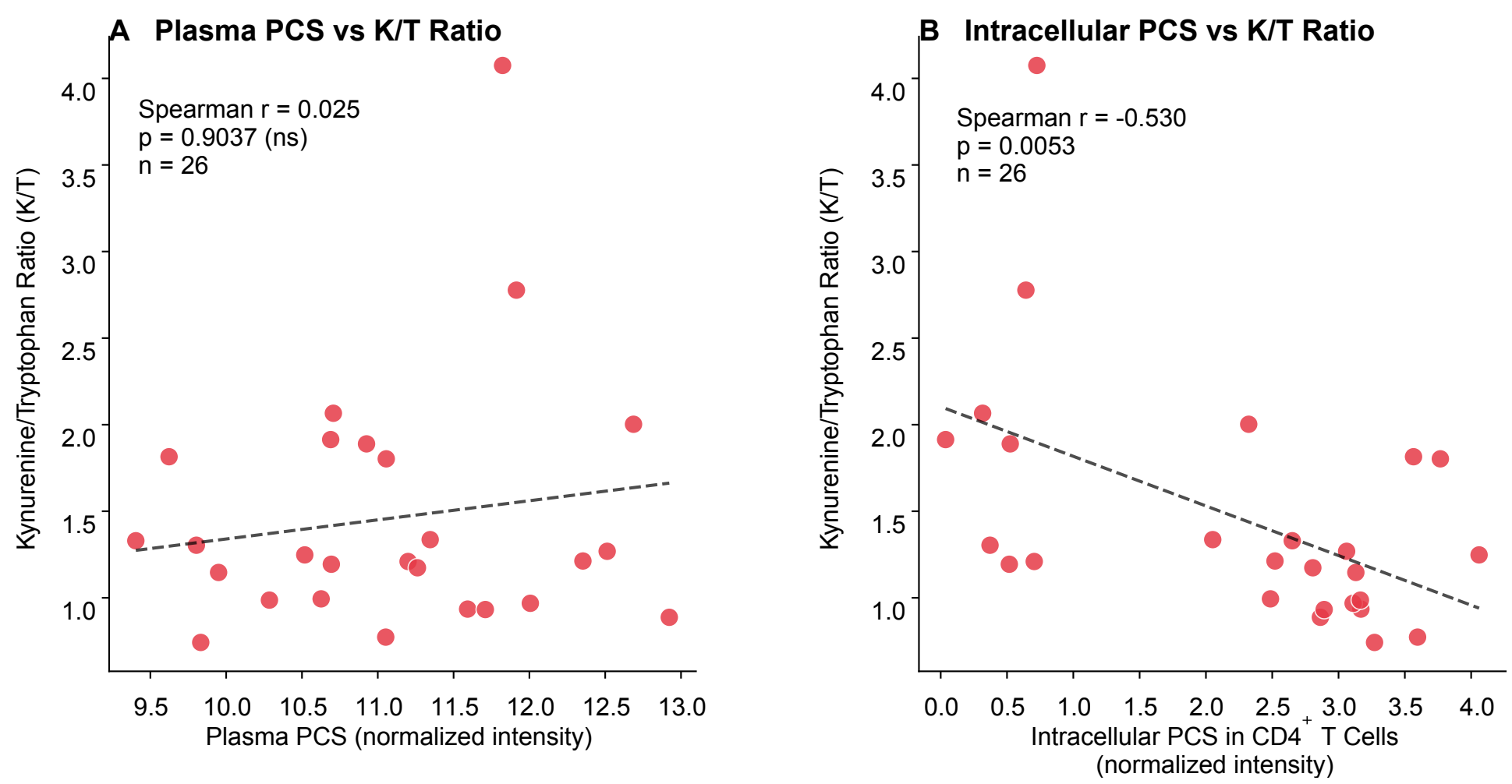

Supplementary Figure 2. Cell-associated but not plasma PCS correlates with the kynurenine/tryptophan (K/T) ratio in PLWH on ART. (A) Spearman correlation between plasma PCS levels and the K/T ratio in the GIR cohort (HIV+ ART-treated individuals, n=26). No significant correlation was observed (r=0.025, p=0.904). **(B)** Spearman correlation between intracellular PCS concentrations in $CD4^+$ T cells and the K/T ratio in the same cohort (n=26). A significant inverse correlation was identified (r=-0.530, p=0.005), demonstrating that cell-associated PCS accumulation is associated with suppression of kynurenine pathway activity. These data further support the biological primacy of the intracellular metabolite compartment over systemic plasma levels.

Supplemental Figure 3

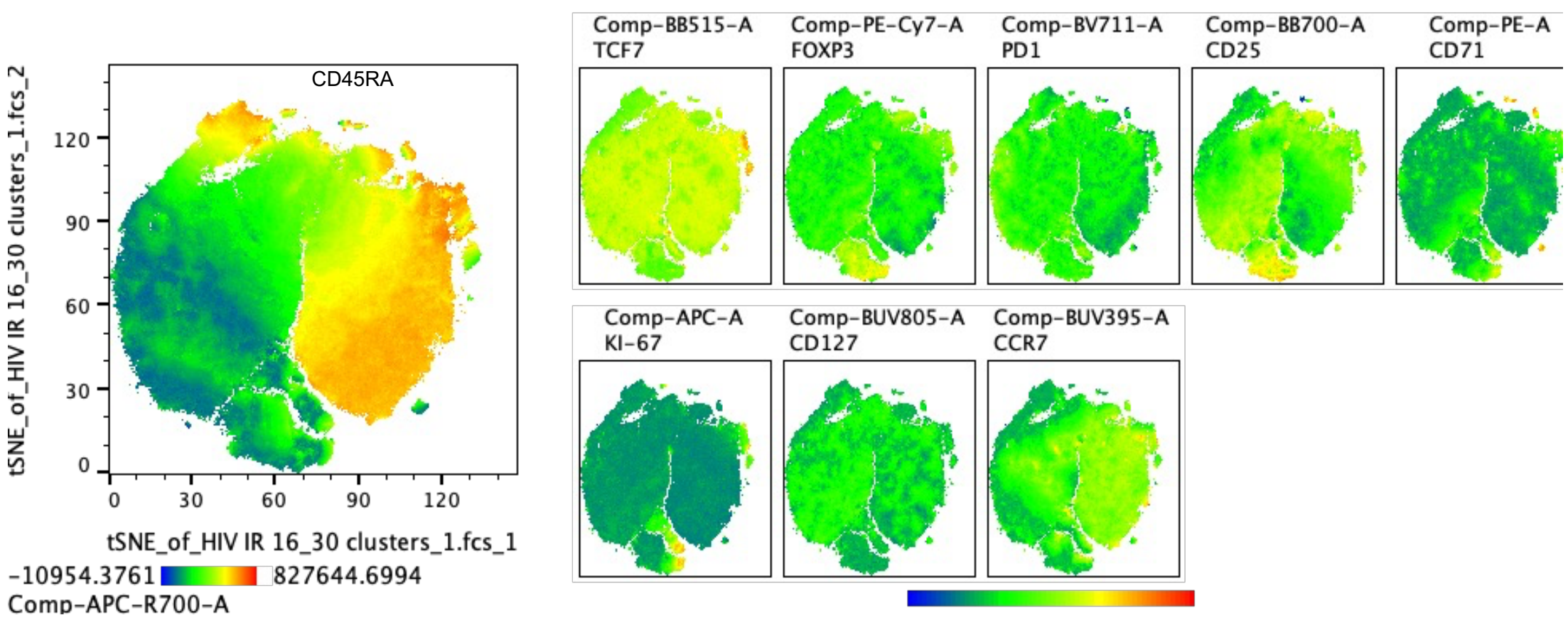

Supplemental Figure 3. Marker distribution across CD4⁺ T-cell clusters used to define populations in Figure 2C. t-SNE projection of CD4⁺ T-cells from PLWH showing single-marker expression intensities across 30 FlowSOM-defined clusters. Each plot represents the expression of the indicated surface or intracellular marker (CD45RA, TCF7, FOXP3, PD-1, CD25, CD71, Ki-67, CD127, CCR7) across the CD4⁺ T-cell landscape. Expression intensities are color-coded from low (blue) to high (red). These marker distributions were used to assign phenotypic identity to CD4⁺ T-cell subsets shown in **Figure 2C**.

Supplementary Figure 4

Healthy controls

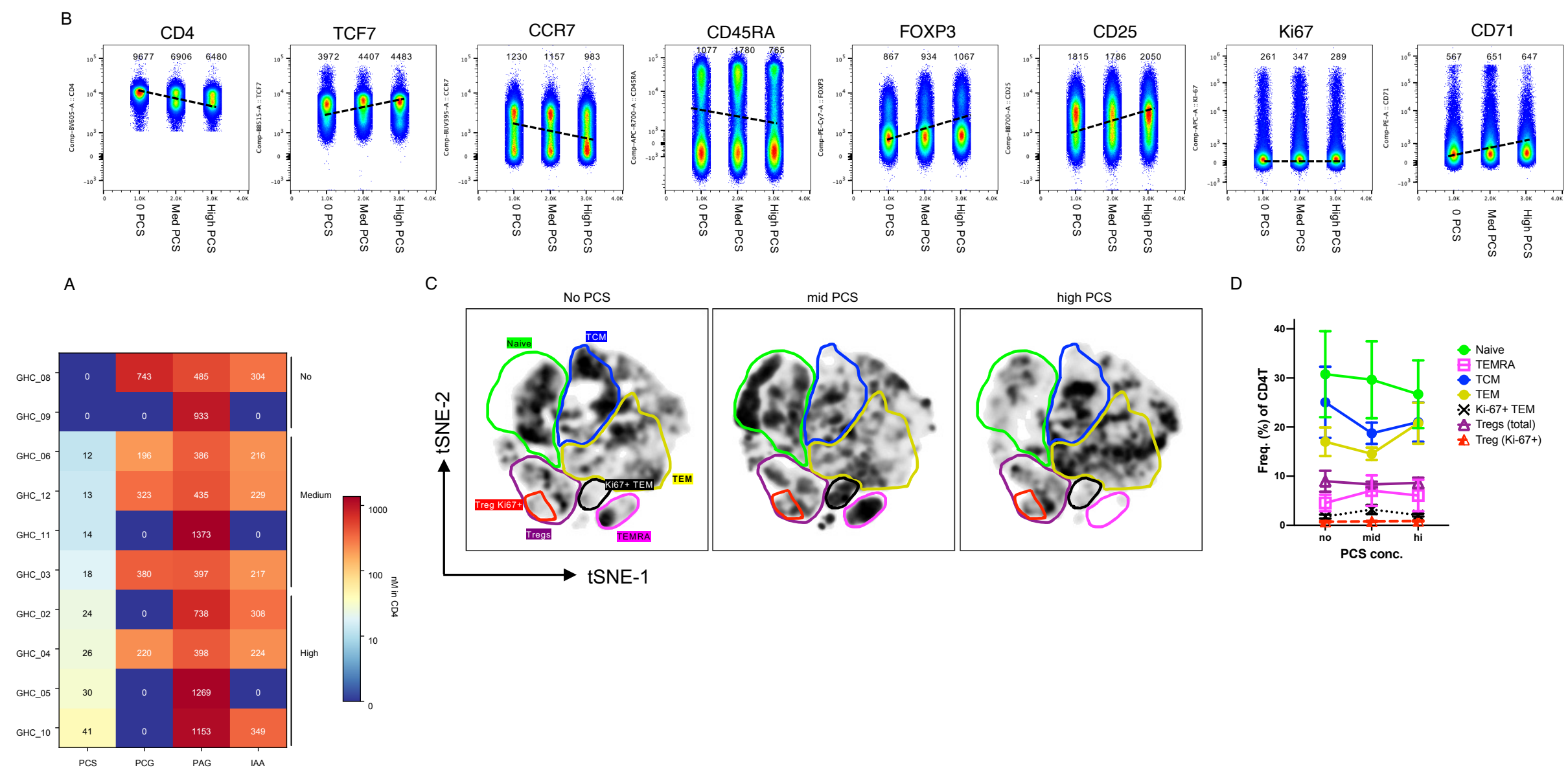

Supplementary Figure 4. Intracellular PCS Accumulation Associates with CD4⁺ T Cell Immunophenotypic Remodeling in Healthy Controls. **(A**) Heatmap showing Intracellular concentrations of PCS, PAG, and IAA measured by LC-MS/MS in CD4⁺ T cells from healthy control donors (GHC) stratified by PCS levels into no (0 PCS), medium, and high intracellular PCS groups. PAG and IAA intracellular concentrations are shown for each donor alongside PCS levels. **(B)** Flow cytometric analysis of CD4⁺ T cells from healthy controls stratified by intracellular PCS concentration (no, medium, and high PCS). Expression of TCF7, CCR7, CD45RA, FOXP3, CD25, Ki67, and CD71 is shown across PCS strata. Dashed lines indicate trend across PCS concentration groups. **(C)** tSNE dimensionality reduction of CD4⁺ T cell flow cytometry data from healthy controls across no PCS, mid PCS, and high PCS conditions, with annotated clusters corresponding to naïve, TCM, TEM, TEMRA, Ki67⁺ TEM, Treg, and TCF7⁺ populations. Progressive remodeling of CD4⁺ T cell subset distribution

is observed with increasing intracellular PCS concentration. **(D)** Quantification of $CD4^+$ T cell subset frequencies (naïve, TEMRA, TCM, TEM, $Ki67^+$ TEM, total Tregs, and $Ki67^+$ Tregs) across no, mid, and high intracellular PCS concentration groups in healthy controls. Data are shown as mean ± SEM. These findings in healthy controls are consistent with the immunophenotypic remodeling observed in PLWH (main manuscript Figure 3), indicating that intracellular PCS-driven $CD4^+$ T cell dysfunction is not exclusive to the HIV disease context and reflects a generalizable relationship between intracellular PCS burden and $CD4^+$ T cell aging.

Supplemental Figure 5

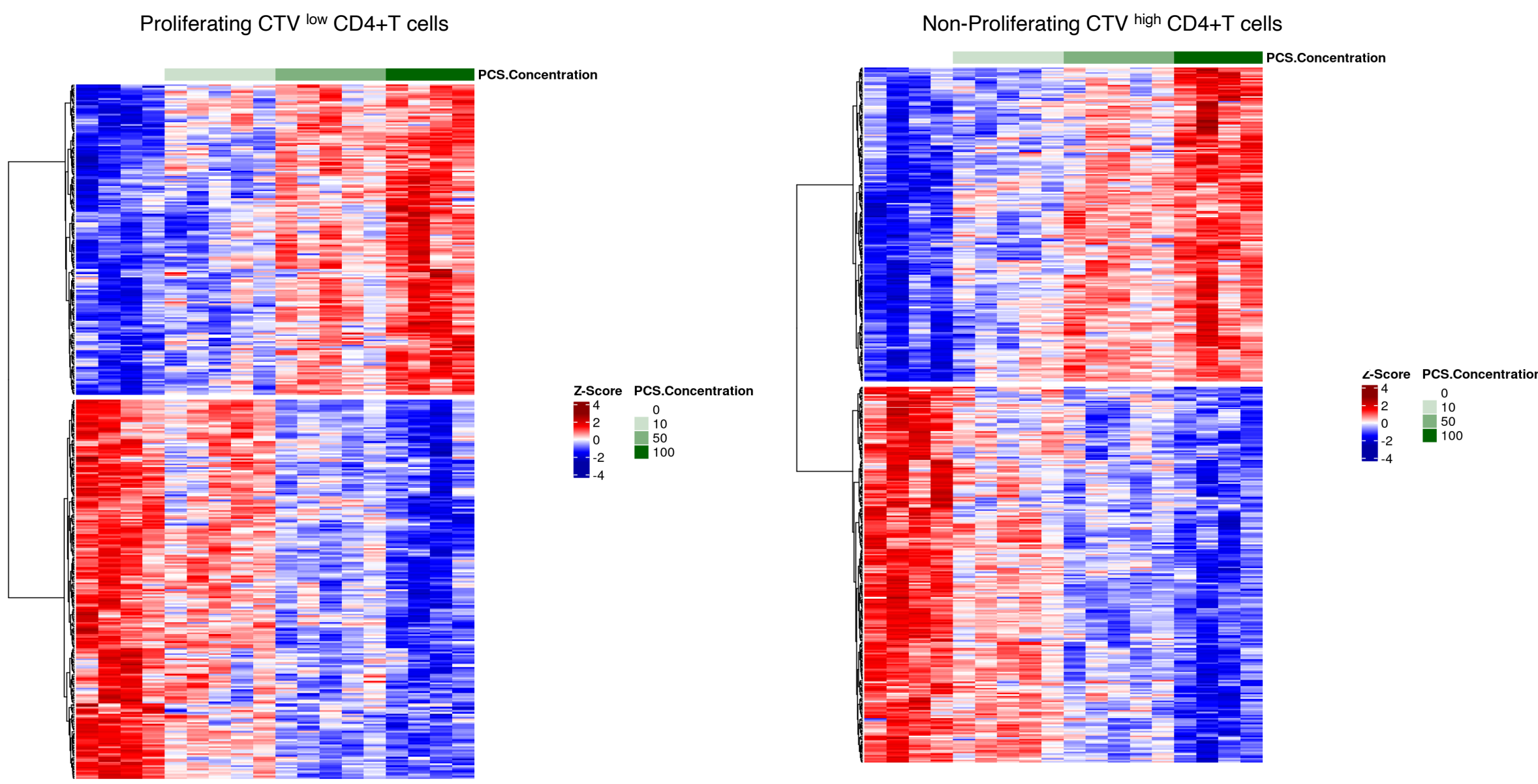

Supplementary Figure 5. Transcriptomic response to PCS in proliferating and non-proliferating CD4⁺ T-cells. Heatmaps show hierarchical clustering of gene expression profiles (Z-score normalized) across PCS doses for proliferating (left) and non-proliferating (right) $CD4^+$ T-cells. Transcriptional changes across both subsets showed broadly similar PCS-induced expression patterns. Based on this similarity, downstream transcriptomic and proteomic analyses were focused on the proliferating (CTV^{low}) $CD4^+$ T-cell subset.

Supplementary Figure 6

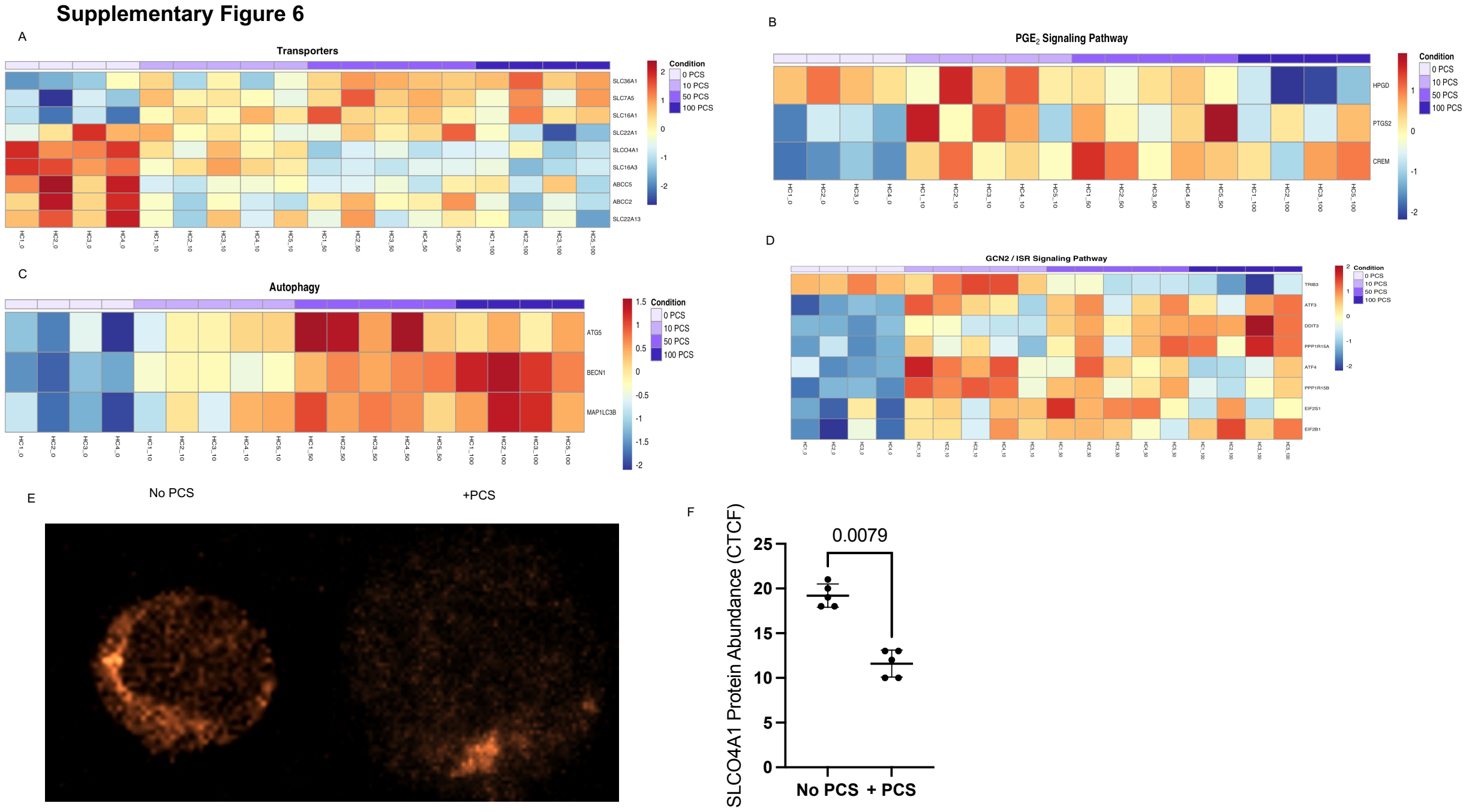

Supplementary Figure 6. PCS Induces Dose-Dependent Transcriptional Reprogramming of Transporter, PGE_2, Autophagy, and GCN2/ISR Pathways and Downregulates SLCO4A1 Protein Expression in Primary $CD4^+$ T Cells. Bulk RNA-seq was performed on primary $CD4^+$ T cells from healthy donors stimulated with anti-CD3/CD28 and exposed to increasing concentrations of PCS (0, 10, 50, and 100 μM) for 6 days. Heatmaps display normalized gene expression ($\log_2$ fold-change relative to vehicle-treated controls) across four mechanistic pathway axes. Each column represents an individual donor-condition replicate; each row represents a gene. Color scale indicates $\log_2$ fold-change with red denoting upregulation and blue denoting downregulation. (A) Transporters: SLCO4A1, encoding OATP4A1, was the sole differentially expressed organic anion influx transporter, showing dose-dependent downregulation. Concurrent downregulation of efflux transporters ABCC2, ABCC5, and SLC22A13 indicates convergent impairment of intracellular metabolite clearance. (B) PGE_2 Signaling Pathway: Dose-dependent upregulation of PTGS2/COX-2 combined with downregulation of HPGD/15-PGDH establishes dual PGE_2 overproduction and impaired degradation, with concurrent CREM upregulation

indicating EP2/EP4–cAMP–PKA axis engagement. (C) Autophagy: Dose-dependent upregulation of ATG5, BECN1, and MAP1LC3B indicates engagement of the canonical autophagy pathway. (D) GCN2/ISR Signaling Pathway: Robust dose-dependent activation of the GCN2/integrated stress response axis evidenced by upregulation of DDIT3/CHOP, ATF3, ATF4, and PPP1R15A. (E) Representative z-stack confocal microscopy images of $CD4^+$ T cells stained for SLCO4A1 protein under control conditions (No PCS, left) and following PCS exposure (+PCS, right). In the absence of PCS, SLCO4A1 displays discrete membrane-localized distribution consistent with its role as a plasma membrane transporter. Upon PCS exposure, SLCO4A1 signal becomes diffuse and dissociates from the cell membrane, indicating loss of membrane-resident protein and internalization or degradation of the transporter. (F) Quantification of SLCO4A1 protein abundance by corrected total cell fluorescence (CTCF) in $CD4^+$ T cells with or without PCS exposure. PCS significantly reduced SLCO4A1 protein abundance ($p = 0.0079$, $n = 5$ donors). Differential gene expression was determined using an FDR-adjusted p value < 0.05 with a minimum $|\log_2$ fold change$| \geq 1$.

Supplementary Figure 7

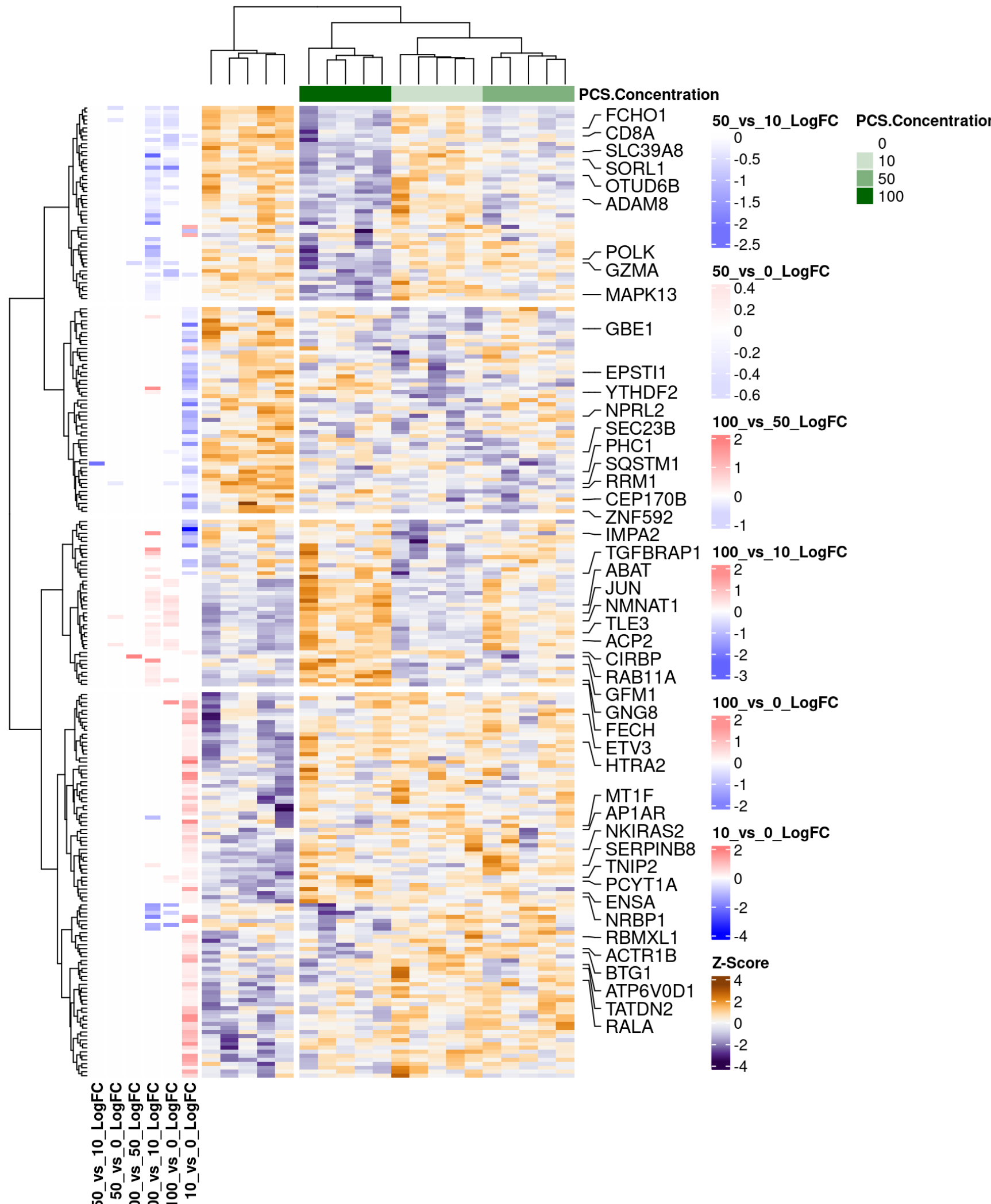

Supplementary Figure 7. PCS Induces a Proteomic Shift from Immune Effector Programs to Mitochondrial and Stress-Adapted States in CD4⁺ T Cells. Global proteomic analysis of primary CD4⁺ T cells exposed to increasing concentrations of PCS (0, 10, 50, and 100 μM) under anti-CD3/CD28 stimulation identified coordinated downregulation of immune effector proteins and upregulation of mitochondrial, metabolic, and stress-adaptive proteins. Downregulated proteins included FCHO1, an adaptor critical for clathrin-mediated endocytosis and TCR internalization; SLC39A8/ZIP8, a zinc transporter required for T cell activation and redox defense; SORL1,

involved in endosomal trafficking and membrane protein recycling; OTUD6B, a deubiquitinase regulating protein stability and immune signaling; ADAM8, a metalloproteinase involved in leukocyte adhesion and migration; GZMA/Granzyme A, a cytotoxic effector molecule; and MAPK13/p38δ, a stress-responsive kinase central to inflammatory signaling and T cell polarization. Upregulated proteins clustered into three functional categories: mitochondrial function and oxidative stress adaptation (GFM1, FECH, HTRA2, RAB11A, GNG8); transcriptional and post-transcriptional regulation (JUN, ETV3, TLE3, CIRBP, TGFBRAP1); and metabolic remodeling and stress buffering (NMNAT1, ACP2, ABAT, IMPA2). Together these findings indicate that PCS suppresses proteins central to immune effector activity and antigen receptor signaling while promoting a compensatory shift toward a metabolically stress-adapted state, consistent with the transcriptional reprogramming and functional suppression reported in the main manuscript. Differential protein abundance was determined using FDR-adjusted p values < 0.05 with a minimum $|\log_2$ fold change$| \geq 1$.

Supplementary Figure 8

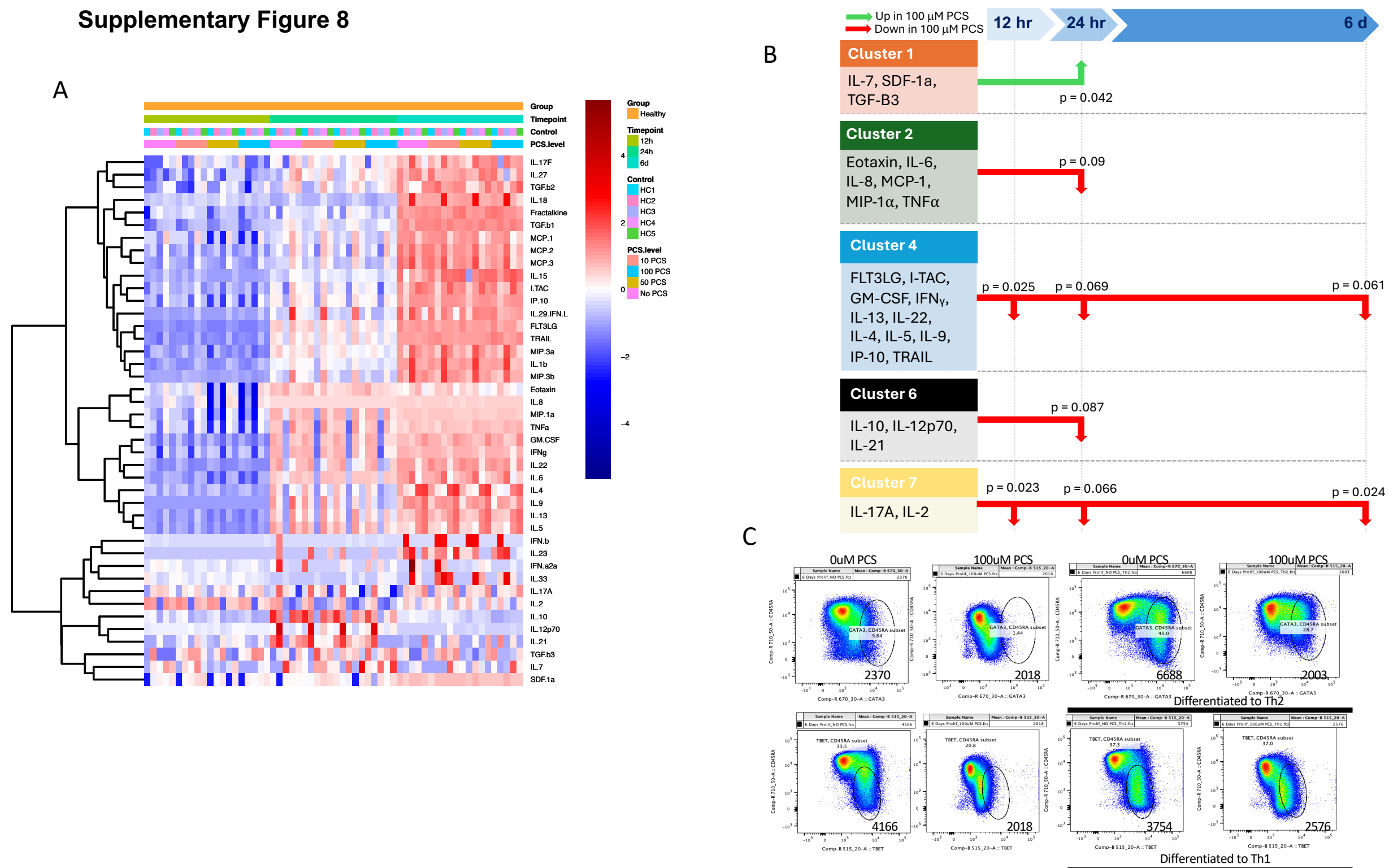

Supplementary Figure 8. PCS modulates cytokine production and impairs Th1/Th2 polarization in CD4⁺ T-cells. (A) Heatmap of 40 cytokines measured in cell culture supernatants by multiplex detection system (MDS) from 5 healthy donors (HC1–HC5). PBMCs were stimulated with anti-CD3/CD28 and cultured in the presence of increasing concentrations of PCS (0, 10, 50, or 100 µM) for 12 hours, 24 hours, or 6 days. Cytokines were hierarchically clustered based on expression patterns. **(B)** Cluster analysis of cytokine profiles identified distinct PCS-responsive modules. Cluster 1 (IL-7, SDF-1a, TGF-β3) was upregulated at 24 hours with 100 µM PCS. In contrast, clusters 2, 4, 6, and 7, which include proinflammatory and T helper–associated cytokines (e.g., IL-6, TNF-α, IL-17A, IL-4, IL-5, IL-13), were significantly downregulated by PCS at multiple

timepoints. Statistical significance determined by paired t tests. **(C)** PBMCs were stimulated with anti-CD3/CD28 for 6 days in the absence or presence of 100 μM PCS under non-polarizing (top left two panels), Th2-polarizing (top right two panels), or Th1-polarizing (bottom four panels) conditions. Cells were gated on $CD4^+$ T-cells and analyzed for the expression of transcription factors GATA3 (top panels) and T-bet (bottom panels). The percentage of positive cells and the corresponding mean fluorescence intensity (MFI) values are shown on each plot. Data in **(C)** are representative of three independent experiments.

Supplementary Table 1

Sample ID	Age	Sex	Race	BMI	Smoker	Last CD4 Count (c/µl)	Time on ART (Years)
GIR 03	60	Male	African American	*29.1*	*No*	612	25.23
GIR 04	63	Female	Caucasian	*28.8*	*No*	671	11.19
GIR 13	57	Female	African American	*32.3*	*No*	1183	6.44
GIR 16	59	Female	African American	*30.4*	*No*	1218	5.04
GIR 22	63	Male	Hispanic	*30.9*	*Yes*	979	11.61
GIR 26	55	Female	African American	*32.3*	*Yes*	700	6.75
**Average**	**59.5**	**4F2M**	**4AF, 1C, 1H**	**30.6**	**2S4NS**	**893.83**	**11.04**
GIR 07	52	Female	Caucasian	*28.4*	*Yes*	1155	5.7
GIR 01	58	Female	Caucasian	*30.4*	*No*	1195	16.38
GIR 23	59	Female	African American	*30.4*	*No*	547	9.65
GIR 09	42	Female	African American	*31.1*	*No*	478	3.4
GIR 05	63	Male	Caucasian	*28.8*	*No*	1150	10.69
GIR 27	59	Female	African American	*30.4*	*No*	567	11.75
**Average**	**55.5**	**5F1M**	**3AF, 3C**	**29.9**	**1S5NS**	**848.67**	**9.595**
GIR 10	55	Male	African American	*32.3*	*Yes*	1004	8.09
GIR 17	40	Female	Hispanic	*29.4*	*Yes*	599	4.32
GIR 11	64	Female	African American	*30.5*	*No*	518	5.34
GIR 25	55	Female	African American	*32.3*	*Yes*	1453	11.73
GIR 20	63	Male	African American	*31.2*	*No*	987	5.81
GIR 12	55	Female	African American	*32.3*	*Yes*	603	11.07
**Average**	**55.33**	**4F2M**	**4AF, 1H**	**31.3**	**4S2NS**	**860.67**	**7.73**
GIR 19	59	Female	African American	*30.4*	*No*	1234	9.55
GIR 21	41	Male	Caucasian	*28.4*	*No*	837	0.2
GIR 15	55	Male	African American	*32.3*	*Yes*	749	10.95
GIR 08	65	Female	African American	*31.1*	*No*	1197	19.03
GIR 02	64	Female	African American	*30.5*	*No*	735	11.41
GIR 24	70	Male	African American	*29.0*	*Yes*	538	7.65
**Average**	**59**	**3F3M**	**5AF, 1C**	**30.3**	**2S4NS**	**881.67**	**9.8**

Supplementary Table 1. Demographic and clinical characteristics of study participants. The table summarizes age, sex, race, last recorded $CD4^+$ T-cell count (cells/µl), and duration of antiretroviral therapy (ART) for all participants of Figure 3. Participants are grouped into four experimental cohorts (n = 6 per group), with averages shown for each cohort. Abbreviations: AF, African American; C, Caucasian; H, Hispanic; ART, antiretroviral therapy

Supplementary Methods

Flow cytometry

Ex vivo and in vitro panels were acquired on a BD FACSymphony A5 and analyzed with FlowJo v10.10.0 (BD Biosciences). The ex vivo panel included: LIVE/DEAD Fixable Near-IR (catalog 501121531, Invitrogen), anti-CD3 BUV737 (clone UCHT1, catalog 612750, BD Biosciences), anti-CD4 BV605 (clone OKT4, catalog 317438, BioLegend), anti-CCR7 BUV395 (clone 2-L1-A, catalog 569108, BD Biosciences), anti-CD45RA R718 (clone HI100, catalog 567073, BD Biosciences), anti-CD127 BUV805 (clone HIL-7R-M21, catalog 748486, BD Biosciences), anti-CD25 BB700 (clone M-A251, catalog 566447, BD Biosciences), anti-p21 AF488 (clone EPR362, catalog ab282187, Abcam), anti-p16INK4a PE (clone EPR1473, catalog ab209579, Abcam). Intracellular staining used the eBioscience FOXP3/Transcription Factor Staining Buffer Set (catalog 00-5523-00, Invitrogen). For in vitro characterization, cells were stimulated for 6 days with anti-CD3/CD28 (200 ng each; BD Biosciences) in RPMI containing 2% human serum and 1% penicillin/streptomycin, with PCS (catalog A8895, APExBio) at 10, 50, or 100 µM. The in vitro panel additionally included anti-Notch1 AF647 (clone MHN1-519, catalog 564782), anti-Notch2 RB780 (clone MHN2-25, catalog 756225), anti-TCF7 BV421 (clone S33-966, catalog 566692), anti-B-catenin AF488 (clone 14/Beta-Catenin, catalog 562505), AhR PE-Cy7 (clone FF3399, catalog 25-9854-42, eBioscience), and CYP1B1 APC (polyclonal, catalog NBP2-97885APC, Novus Biologicals), with appropriate isotype controls (BD Biosciences).

Th1/Th2/Treg Differentiation

Naïve $CD4^+$ T cells were isolated from healthy donor PBMCs using the EasySep Human Naïve $CD4^+$ T Cell Isolation Kit II (catalog 17555, STEMCELL Technologies) and stimulated for 6 days with T Cell TransAct (1/3 recommended concentration; catalog 130-128-758, Miltenyi Biotec) in AIM V medium with Nu-Serum IV supplement in the presence of graded PCS concentrations. Th1 differentiation used IL-12 (10 ng/mL; PeproTech) and anti-IL-4 (2.5 µg/mL; Miltenyi Biotec); Th2

used IL-4 (10 ng/mL; PeproTech) and anti-IFNγ (1 μg/mL; Miltenyi Biotec). The readout panel included anti-T-bet AF488 (clone 4B10, catalog 568177), anti-Eomes PE (clone X4-83, catalog 566749), anti-GATA3 AF647 (clone L50-823, catalog 560068), and TCF7 BV421 (clone S33-966, catalog 566692; all BD Biosciences), alongside the backbone markers listed above.

Cytokine Profiling

Cytokines were measured in cell culture supernatants using a U-PLEX multiplex assay (Meso Scale Diagnostics, Rockville, MD) on a MESO QuickPlex SQ 120 reader. Results were reported in pg/mL and extrapolated from analyte-specific standard curves.

Single-cell RNA-seq

Frozen PBMCs were thawed and $CD4^+$ T cells enriched using the EasySep Human $CD4^+$ T Cell Enrichment Kit (StemCell Technologies). Single-cell libraries were prepared using the Chromium Next GEM Single Cell 5' Reagent Kit v2 (10x Genomics) targeting 10,000 cells per sample, and sequenced on an Illumina NovaSeq S4 using 10x-recommended parameters. Raw and processed data are deposited in GEO under accession GSE328611.

Bulk RNA-seq

$CD4^+$ T cells from healthy donors were labeled with CellTrace Violet (catalog C34557, Invitrogen), stimulated with anti-CD3/CD28 for 6 days in the presence of PCS (10, 50, or 100 μM; APExBio), and proliferating cells sorted on a BD FACSAria II. RNA was isolated using the RNeasy Kit (QIAGEN) and libraries prepared with the Illumina Stranded Total RNA Prep with Ribo-Zero Plus Kit. Sequencing was performed on an Illumina NovaSeq S4. Raw reads were aligned to GRCh38 using STAR. Raw and processed data are deposited in GEO under accession GSE329693.

Proteomics Sample Preparation and LC-MS/MS

Cell pellets were resuspended in RAD buffer (100 mM Tris pH 8.8, 1% SDC, 10 mM TCEP, 40 mM CAA), boiled at 95°C for 10 minutes, and digested overnight at 37°C with Trypsin/Lys-C (1:40 w/w; Promega). Peptides were acidified, cleaned on Oasis HLB plates (Waters), and quantified by Pierce Colorimetric Peptide Assay (Thermo Fisher Scientific). 200 ng per sample was injected

on a Bruker NanoElute coupled to a timsTOF Pro2 (Bruker Daltonics) and acquired in diaPASEF mode. Protein identification and quantification used Spectronaut in library-free mode against the SwissProt human database, with carbamidomethylation (C) as fixed and acetylation (N-term)/oxidation (M) as variable modifications. Raw and processed data are deposited in ProteomeXchange/PRIDE under accession PXD077610.

Metabolic Analysis

Untargeted and targeted metabolic analyses were performed at the Clinical Biomarkers Laboratory, Emory University School of Medicine. For untargeted metabolomics, cells and plasma were extracted with ice-cold 80:20 acetonitrile:water and analyzed in triplicate on a Thermo Ultimate3000 UHPLC–Q Exactive HF system using parallel HILIC/ESI+ and C18/ESI− platforms at 120k resolution. Feature extraction used apLCMS (v6.3.3) with xMSanalyzer quality control; features were annotated against the Human Metabolome Database using xMSannotator. For targeted quantification of PCS, PCG, PAG, and IAA, standard addition was performed on a Vanquish Duo UHPLC coupled to an Orbitrap ID-X (Thermo Fisher Scientific) with MS2 confirmation at 35% HCD. Raw and processed metabolomics data are deposited in the Metabolomics Workbench (Study ID ST004747; DOI: dx.doi.org/10.21228/M86V8D).

Confocal microscopy

$CD4^+$ T cells from healthy donors were isolated from PBMCs using the EasySep Human $CD4^+$ T Cell Enrichment Kit (StemCell Technologies) and stimulated with anti-CD3/CD28 for 6 days in the presence or absence of 100 μM PCS. Cells were then collected, washed in PBS, and cytospun onto poly-L-lysine coated glass slides. Cells were fixed with 4% paraformaldehyde for 15 minutes at room temperature, permeabilized with 0.1% Triton X-100 for 10 minutes and blocked with 5% normal goat serum in PBS for 1 hour. Slides were incubated overnight at 4°C with a primary polyclonal antibody against SLCO4A1 (ThermoFisher #PA5-56535), followed by washing and incubation with a fluorescent secondary antibody for 1 hour at room temperature protected from light. Nuclei were counterstained with DAPI. Coverslips were mounted using ProLong Gold

Antifade Mountant (Thermo Fisher Scientific). Z-stack images were acquired on Nikon A1R confocal microscope (Nikon Instruments Melville, New York, USA). For each condition, 5 fields were imaged. SLCO4A1 protein abundance was quantified as corrected total cell fluorescence (CTCF) using ImageJ (NIH), calculated as: CTCF = Integrated Density − (Area of selected cell × Mean fluorescence of background readings). Statistical comparison between No PCS and +PCS conditions was performed using a paired t-test.

HIV Provirus Detection

Intact and total proviral DNA were quantified by IPDA (Accelevir Diagnostics, Baltimore, MD).
